# Infection prevention and care bundles addressing health care-associated infections in neonatal care in low-middle income countries: a scoping review

**DOI:** 10.1016/j.eclinm.2021.101259

**Published:** 2022-01-10

**Authors:** Alexandra Molina García, James H. Cross, Elizabeth J.A. Fitchett, Kondwani Kawaza, Uduak Okomo, Naomi E. Spotswood, Msandeni Chiume, Veronica Chinyere Ezeaka, Grace Irimu, Nahya Salim, Elizabeth M. Molyneux, Joy E. Lawn, with the NEST360 Infection Prevention, Detection and Care Collaborative Group

**Affiliations:** a MARCH Centre, London School of Hygiene and Tropical Medicine, London, UK; b UCL Great Ormond Street Institute of Child Health, London, UK; c Department of Paediatrics, Kamuzu University of Health Sciences (formerly College of Medicine, University of Malawi), Blantyre, Malawi; d Medical Research Council Unit, The Gambia at London School of Hygiene and Tropical Medicine, Fajara, The Gambia; e Maternal, Child and Adolescent Health Program, Burnet Institute, Melbourne, VIC, Australia; f Department of Paediatrics, College of Medicine, University of Lagos, Nigeria; g Department of Paediatrics and Child Health, University of Nairobi, Kenya; h Department of Paediatrics and Child Health, Muhimbili University of Health and Allied Sciences, Dar Es Salaam, Tanzania

**Keywords:** scoping review, health care-associated infections, care bundles, neonatal units, infection prevention and control, low- and middle-income countries, 3 + I Framework, (1) Primary Prevention (2) Detection (3) Case Management + Implementation, ERIC, Expert Recommendations for Implementing Change, HCAI, Health Care-Associated Infections, LMIC, Low- and Middle-Income Countries, PRISMA, Preferred Reporting Items for Systematic Reviews and Meta-Analyses, PRISMA-ScR, Preferred Reporting Items for Systematic Reviews and Meta-Analyses for Scoping Reviews

## Abstract

**Background:**

Health care-associated infections (HCAI) in neonatal units in low- and middle-income countries (LMIC) are a major cause of mortality. This scoping review aimed to synthesise published literature on infection prevention and care bundles addressing neonatal HCAI in LMICs and to construct a Classification Framework for their components (elements).

**Methods:**

Five electronic databases were searched between January 2001 and July 2020. A mixed-methods approach was applied: qualitative content analysis was used to build a classification framework to categorise bundle elements and the contents of the classification groups were then described quantitatively.

**Findings:**

3619 records were screened, with 44 eligible studies identified. The bundle element Classification Framework created involved: (1) *Primary prevention,* (2) *Detection*, (3) *Case management*, and *Implementation* (3 + I*)*. The 44 studies included 56 care bundles with 295 elements that were then classified. *Primary prevention* elements (128, 43%) predominated of which 71 (55%) focused on central line catheters and mechanical ventilators. Only 12 elements (4%) were related to *detection*. A further 75 (25%) elements addressed *case management* and 66 (88%) of these aimed at outbreak control.

**Interpretation:**

The 3 + I Classification Framework was a feasible approach to reporting and synthesising research for infection-relevant bundled interventions in neonatal units. A shift towards the use in infection prevention and care bundles of *primary prevention* elements focused on the neonate and on commonly used hospital devices in LMIC (e.g., self-inflating bags, suctioning equipment) would be valuable to reduce HCAI transmission. *Detection* elements were a major gap.

**Funding:**

This work was made possible in part by the John D. and Catherine T. MacArthur Foundation, the Bill & Melinda Gates Foundation, ELMA Philanthropies, The Children's Investment Fund Foundation UK, The Lemelson Foundation, and the Ting Tsung and Wei Fong Chao Foundation under agreements to William Marsh Rice University. The project leading to these results has also received the support of a fellowship from the “la Caixa” Foundation (ID 100010434). The fellowship code is LCF/BQ/EU19/11710040. EJAF is an Academic Clinical Fellow whose salary is funded by the UK National Institute for Health Research (NIHR). NES receives a Research Training Program Scholarship (Australian Commonwealth Government).

**Research in context**

**Evidence before this study**

Health care-associated infections (HCAI) are a major cause of neonatal morbidity and mortality in low- and middle-income countries (LMIC) and of increasing concern owing to their associated antimicrobial resistance. Infection prevention and care bundles have expanded to neonatal care settings and proven effective in reducing HCAI. Consequently, the creation of a Classification Framework for the care bundles’ components (elements) will provide a basis for a uniform nomenclature for the creation of future care bundles. EMBASE, Pubmed, Global Health, CINAHL, and Web of Science were searched for studies published between January 2001 and July 2020 with the search terms: “neonate”, “care bundles”, “health care-associated infections”, and “low- and middle-income countries”.

**Added value of this study**

This is the first scoping review synthesising all published literature on neonatal infection prevention and care bundles addressing HCAI in LMIC. From 56 bundles 295 individual elements were identified. A novel 3 + I Classification Framework was created into which these elements were classified, covering: (1) *Primary prevention,* (2) *Detection,* (3) *Case management* + *Implementation* (3 + I*)*. Almost half the bundle elements were for infection *primary prevention,* notably targeting central line catheters and mechanical ventilators. Importantly we found almost a total lack of elements aimed at HCAI *detection. Case management* elements focused on the supportive care of neonates with HCAI were scarce.

**Implications of all the available evidence**

The 3 + I Classification Framework provided a systematic way to organise the elements of infection prevention and care bundles. Further research is required on infection *detection* elements in care bundles. We highlight the need for innovations in detection and surveillance systems, especially in LMIC where laboratory services are limited yet the burden of HCAI is highest. This *detection* gap could be further simplified with advances in point-of-care testing.

Alt-text: Unlabelled box

## Introduction

Around the world, an estimated 2.4 million neonates die every year. Almost 80% of these deaths occur in sub-Saharan Africa and Southern Asia.^1^ The Sustainable Development Goals set by the United Nations include a target to reduce national neonatal death rates to less than 12 per 1000 live births by 2030.^2^ To deliver this target, a higher coverage and quality of health care is needed for the 30 million newborns requiring hospital care annually.^3^

The emergence of antimicrobial resistance associated with the widespread but often unreported health care-associated infections (HCAI) is a significant threat to progress for ending preventable neonatal deaths.^4^^,^^5^ In low- and middle-income countries (LMIC) the HCAI incidence in inpatient newborn care units is estimated to be 15.2 to 62.0 per 1000 patient-days; nine times higher than observed in some high-income settings.^6^ Infection prevention and control interventions need to be implemented into daily neonatal care to reduce neonatal mortality and improve health care quality.

Care bundles are a strategy developed by the Institute for Healthcare Improvement to strengthen the quality of care in adult intensive care units in 2001.^7^ Each care bundle is a collection of evidence-based practices (called bundle ‘elements’) implemented together to improve patient outcomes. The use of infection prevention and care bundles has rapidly extended to neonatal care settings and proven effective in reducing adverse clinical outcomes, including HCAI.^8^, ^9^, ^10^ To provide consistency in their formulation and implementation for reducing neonatal HCAI in LMIC, a classification system is needed. The creation of a Classification Framework will also provide common and consistent terminology and definitions for the care bundle element categories. These element categories can be used by neonatal health services as potential building blocks for the construction of infection prevention and care bundles, proposing a holistic approach in their design to address the different causes (e.g., contact transmission through health care staff, lack of hand hygiene equipment, or inappropriate use of antibiotics) and stages of infection (e.g., prevention, detection, or control).^11^, ^12^, ^13^ This taxonomy will also highlight evidence gaps for future research to tackle HCAI.

Previous systematic reviews on care bundles in neonatal settings have focused on evaluating their effectiveness in reducing central line- or ventilator-associated infections.^8^^,^^9^ However, no published review has assembled the infection prevention and care bundles addressing neonatal HCAI in LMIC, and there is no existing framework to categorise them and their care bundle elements.

This scoping review aimed to search and synthesise published literature on infection prevention and care bundles addressing neonatal HCAI in LMIC. The objectives were to build a bundle element Classification Framework based on identified care bundles and to quantitatively analyse its content.

## Methods

**Figure 1:**
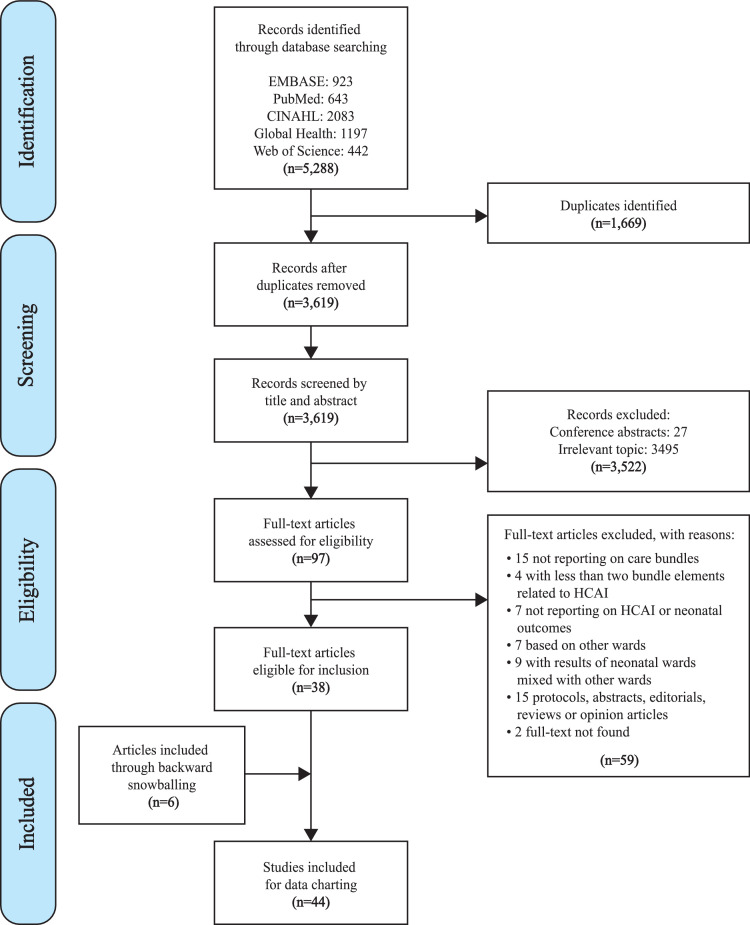
PRISMA flow diagram of study selection. **Abbreviations:** n = number of records; HCAI = health care-associated infections.

This scoping review was based on the guidance framework for conducting scoping reviews developed by the Joanna Briggs Institute.^14^ It is reported using the Preferred Reporting Items for Systematic Reviews and Meta-Analyses Extension for Scoping Reviews (PRISMA-ScR).^15^ The study protocol was registered with the Open Science Framework (Fig. 1 in Appendix).^16^

### Information sources and search strategy

The literature search was performed across the databases EMBASE, Pubmed, Global Health, CINAHL, and Web of Science. The search strategy included English keywords and medical subject headings for four concepts: neonates, care bundles, HCAI, and LMIC (Table 1 in Appendix). In 2001, the Institute for Healthcare Improvement developed the concept of care bundles,^7^ therefore, searches were limited to studies published from 2001 until July 3rd, 2020 in English, Spanish, and French languages.

### Eligibility criteria

The main inclusion criteria were aligned with the PCC mnemonic (Population, Concept, Context), as follows (Table 2 in Appendix): For the first mnemonic term (‘Population’), studies were included if neonates (infant less than 28 days of life) were the target study population. Concerning the second term (‘Concept’), studies were eligible for inclusion if reporting on: (1) care bundles (as defined by the Institute for Healthcare Improvement as: "A small set of evidence-based interventions for a defined patient segment/population and care setting that, when implemented together, will result in significantly better outcomes than when implemented individually")^7^ and (2) any measures of HCAI disease frequency, exposure effects, or any neonatal outcomes. To meet this second inclusion criteria, care bundles had to incorporate at least two elements related to HCAI prevention, detection, control, or management after birth. Thirdly, included studies were set in inpatient newborn care units (all levels of care) in LMIC (World Bank, 2020) (‘Context’).^17^

**Table 2:** Qualitative data summary findings of the 3 + I Classification Framework.

**a. Groups identified**	

**Name**	**Description**

Primary prevention	Elements aiming to avoid health care-associated infections in neonatal care units (e.g., promotion of kangaroo mother care or breastfeeding, reinforcement of the staff's hand hygiene).

Detection	Elements focused on secondary prevention such as the screening and surveillance of health care-associated infections in infected newborns admitted in inpatient neonatal care units (e.g., implementation and reinforcement of infection surveillance programmes).

Case management	Elements focused on tertiary prevention. They describe care of infected neonates or interventions to control the propagation of health care-associated infections in the neonatal units (e.g., cohorting of neonates, changes in antibiotic policy and stewardship, improvement to environmental and equipment disinfection protocols).

Implementation	Elements directed towards the methods of enhancing the adoption, implementation, or sustainability of interventions (e.g., provision of single use fluid vials or alcohol-based hand rub, conduct educational meetings on infection prevention and control measures, establishing audit and feedback mechanisms).

**b. Subgroups identified**	

**Name**	**Description**

Neonate	Elements aimed directly at the neonate.

Staff	Elements aimed at the health care staff.

Caretaker	Elements focused on the caretakers of the admitted newborn patients.

Environment	Elements directed towards the surroundings of the neonate in the inpatient neonatal care units and its organisation.

Device	Elements tackling nosocomial infections acquired through medical equipment.

Screening	Elements that encompass the detection of disease outbreaks and their risk factors.

Epidemiological surveillance	Bundle elements focused on the collection, analysis, and monitoring of data on health care-associated infections in neonatal care units.

Antibiotic prescription	Elements aimed at implementing or improving antibiotic policy and stewardship of inpatient neonatal care settings.

Outbreak control	Elements focused on controlling infectious outbreaks detected in the inpatient neonatal care units.

Audit and feedback	Elements focused on collecting clinical performance data to share with neonatal health care staff and managers to monitor, evaluate, and modify their behaviour*.

Change physical structure and equipment	Bundle elements that evaluate the existing set-up of the neonatal wards and adapt their physical structure and/or equipment to improve the quality of care*.

Conduct educational meetings	Bundle elements that teach all the interested groups about the health care intervention implemented in the neonatal ward through meetings*.

Create or change credentialing and/or licensure standards	Elements aiming at creating or changing a system that certifies staff's skills in the health care intervention and/or grants the health care system or unit with a license to implement an intervention*.

Create new clinical teams	Interventions that change health care staff members to ensure that the health care intervention is delivered by incorporating new skills and work profiles to the team*.

Develop educational materials	Elements focused on the creation of unit protocols, guidelines, tools, manuals, or other materials to improve staff's training and understanding of the health care innovation*.

Organise clinician implementation team meetings	Bundle elements that establish meetings for the clinicians responsible for implementing the health care intervention to ensure a time for reflection on the implementation process and for sharing lessons learnt*.

Recruit, designate and train for leadership	Elements that enrol, assign, and train the leaders of the clinical innovation in the neonatal units*.

Remind clinicians	Elements directed at creating reminder systems to promote the use of or provide information on a health care intervention in the neonatal wards*.

Revise professional roles	Bundle elements aiming at reviewing and changing the job profiles and responsibilities of the neonatal health care staff*.

Table describing the different groups (a) and subgroups (b) identified after performing the qualitative data analysis to construct the Classification Framework for care bundle elements. If bundle elements were coded with headings related to implementation strategies, these were grouped after the categories created by Powell and colleagues in the Expert Recommendations for Implementing Change (ERIC) study.^19^**Legend**: *Adaptation of the category definitions proposed by Powell and colleagues in the Expert Recommendations for Implementing Change (ERIC) study to the neonatal care units.^19^

All study designs were included. Exclusion criteria were: studies set outside of newborn care wards, studies with results including infants older than 28 days, studies reporting on guidelines, single interventions, management protocols, conference abstracts, editorials, reviews, research protocols, opinion articles, or publications where the full-text could not be accessed.

### Selection of sources of evidence

Duplicates were removed from the identified records. In the first stage, all studies were screened by one reviewer (AMG) by title and abstract. A second reviewer (EJAF), who was blinded to the screening results of the first reviewer, screened a random sample of 20 of these studies, with 100% agreement between reviewers. In the second stage, all full-text articles were assessed for eligibility independently by both reviewers, with 93% agreement on the articles to be included. The disagreements in this stage were resolved by consensus between the two reviewers.

### Data charting process

T2he data charting form was piloted by AMG and EJAF on two articles. The following data were extracted by AMG: study characteristics (i.e., first author, year of publication, aim, country of study, study design, level of inpatient newborn care units and terminology found to describe the care bundles). The number and description of bundle elements were extracted independently by both reviewers, blinded to each other's findings. Both reviewers agreed on their identification on 73% of studies. The bundle elements of the rest of the studies were resolved by consensus between reviewers. Within included studies, any bundle elements implemented in settings other than inpatient newborn care units (i.e., labour ward) were not included.

### Synthesis of results and methodology to construct the Classification Framework

**Figure 2:**
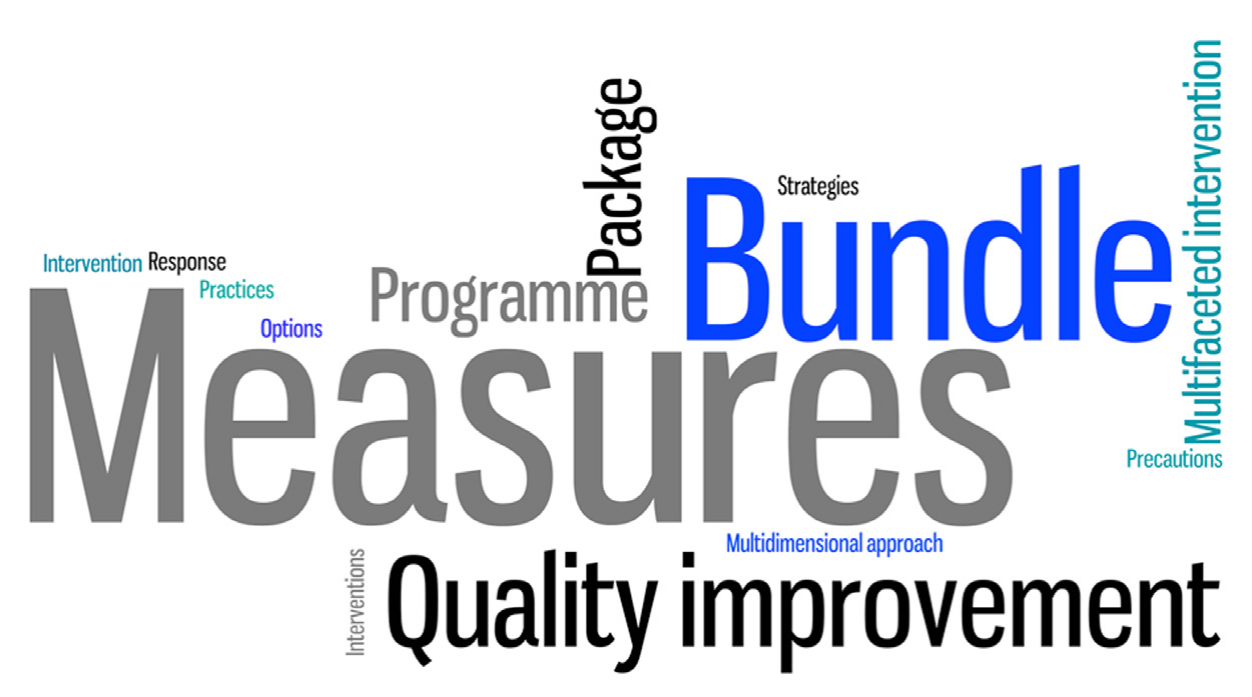
Word cloud with the terminology used to name the care bundles. The world cloud visually represents the names used to describe care bundles depicted in different sizes based on the frequency of their use in the 44 included studies: the higher the frequency of a name, the bigger its appearance in the cloud. Frequencies of the names: Measures = 13 (30%); Bundle = 9 (21%); Quality Improvement = 5 (11%); Programme = 4 (9%); Package = 3 (7%); Multifaceted Intervention = 2 (5%); Precautions = 1 (2%); Strategies = 1 (2%); Practices = 1 (2%); Options = 1 (2%); Multidimensional Approach = 1 (2%); Response = 1 (2%); Intervention = 1 (2%); Interventions = 1 (2%).

The synthesis was displayed narratively, including qualitative and quantitative analysis. Qualitative inductive content analysis was carried out to build the Classification Framework for the bundle elements. An inductive approach was used as no previous care bundle element Framework was identified in the literature. It followed the three-step process proposed by Elo and colleagues: preparation, organisation and reporting (Figure 2 in Appendix).^18^ In the preparation step, care bundle elements (i.e., the single interventions composing a care bundle) were extracted from the bundles, exported, and read multiple times. In the organisation step, each bundle element was labelled with a coding heading to summarise their meaning. Coding headings that shared similar meaning were collated under higher order headings for the development of groups and subgroups. Following this, each group and subgroup was named based on the information it contained and was described narratively to provide a definition. If bundle elements were coded with headings related to implementation strategies, these were grouped after the categories created by Powell and colleagues in the Expert Recommendations for Implementing Change (ERIC) study.^19^ To further synthesise the results, once the bundle elements were grouped, whole bundles were also categorised according to the groups their elements were classified into. In the reporting step, descriptive and quantitative analyses (frequencies and percentages) of the content of the groups and subgroups of the Classification Framework were performed. This analysis was conducted by AMG and supported by JHC and JEL in the organisation phase if doubts arose concerning bundle element labelling, to reach consensus.

The focus of the review was to explore the breadth of the infection prevention and care bundles present in the literature for neonatal settings in LMIC and to map their elements in a Framework. Therefore, a critical appraisal of the studies was not performed as analysing the outcomes concerning infection prevention and care bundles was outside of the scope of the review.

### Statistical analysis

Qualitative analysis was performed to construct the Classification Framework using Microsoft Excel (Microsoft, Redmond WA, USA). Quantitative analysis using frequencies and percentages were carried out for the synthesis of the results. No statistical tests were performed.

### Ethics statement

The Research Ethics Committee at the London School of Hygiene and Tropical Medicine assessed this research project as not requiring ethical approval (Reference: 21720).

### Role of funding sources

Funding agencies had no contributions to the study design, data collection, data analysis, data interpretation, writing of the manuscript, or the decision to submit the paper. These agencies had no access to the dataset of this study. AMG, JHC, EJAF and JEL had access to the study dataset. AMG, JHC and JEL decided to submit the study for publication.

## Results

A total of 5288 publications were identified (Figure 1). After duplicate removal, 3619 records remained for screening. After screening these records by title and abstract, 97 publications were selected for full-text screening. 59 of these publications were excluded according to the eligibility criteria, and the remaining 38 articles were selected for inclusion. Six other articles that fulfilled the inclusion criteria were identified through a backward snowballing technique of the 38 articles. Overall, 44 studies were included for analysis (Table 1).

**Table 1:** Characteristics of the 44 included studies.

Author (Year)	Countryα			Aim of Study	Study Design	Level of INCU	Number of Care Bundles	Number of Bundle Elements
**Type 1 - Primary Prevention Bundles**								

Arora^20^ (2019)	India			Evaluate the impact of management guidelines on neonatal morbidity and mortality of VLBW neonates	Uncontrolled, before-after	III	3	CL insertion bundle: 7 CL maintenance bundle: 6 CL hub care bundle: 5
Azab^37^ (2015)	Egypt			Evaluate the effectiveness of VAP prevention bundle on rates of neonatal VAP	Uncontrolled, before-after	III	1	7
Balla^21^ (2018) β	India			Reduce neonatal CLABSI rates by 25% in three months and to sustain this over the next nine months	Uncontrolled, before-after	III	1	CL removal bundle: 2
Hussain^33^ (2020)γ	Pakistan			Design a CLABSI prevention package to decrease CLABSI rates	Uncontrolled, before-after	III	1	CL maintenance bundle: 5
Resende^34^ (2011)	Brazil			Reduce CLABSI rates using a care bundle	Uncontrolled, before-after	III	1	5
Resende^35^ (2015)	Brazil			Evaluate the impact of an evidence-based bundle in LOS incidence rates	Uncontrolled, before-after	III	1	7
Rosenthal^36^ (2013)δ	El Salvador, Mexico, The Philippines, and Tunisia			Evaluate the impact of INICC multidimensional infection control programme to reduce CLABSI	Uncontrolled, before-after	III	1	CL: 7
Tran^38^ (2018)	Vietnam			Evaluate the impact of the EENC on clinical practices, NICU admissions, and adverse newborn outcomes	Uncontrolled, before-after	III	1	8
Wang^27^ (2015)	China			Evaluate the effectiveness and feasibility of a CL bundle guideline with a standard checklist in the prevention of PICC-related infections in VLBW infants	Uncontrolled, before-after	III	2	CL insertion bundle: 5 CL maintenance bundle: 4

**Type 2 - Detection Bundles**								

No detection bundles in the studies								

**Type 3 - Case Management Bundles**								

Bouallègue-Godet^57^ (2004)	Tunisia			Report outbreak of *S. enterica serotype Livingstone* resistant to extended-spectrum cephalosporins Perform molecular subtyping	Retrospective, descriptive	III	1	3
Indarso^58^ (2008)	Indonesia			Report outbreak of *S. worthington* Report measures to control outbreak	Retrospective, descriptive	NR	1	5
Jeena^49^ (2001)	South Africa			Report outbreak of *A. anitratus* Determine the cause, source, and modes of transmission of the outbreak Report measures to control outbreak	Retrospective, descriptive	III	1	7
Lithgow^51^ (2009)	Papua New Guinea			Report outbreak of *K. pneumoniae* Determine the cause and source of the outbreak Report measures to control outbreak	Retrospective, descriptive	NR	1	4
Moodley^12^ (2005)	South Africa			Report outbreak of *K. pneumoniae* Determine the cause and source of the outbreak	Retrospective, descriptive	III	1	3
Shanmuganathan^22^ (2004)	India			Report outbreak of *K. pneumoniae*	Retrospective, descriptive	III	1	3

**Type 4 - Implementation Bundles**								

Balla^21^ (2018) β	India			Reduce neonatal CLABSI rates by 25% in three months and to sustain this over the next nine months	Uncontrolled, before-after	III	1	CL insertion bundle: 3
Cavicchiolo^44^ (2016)ε	Mozambique			To assess the effectiveness of interventions in terms of reduction of the neonatal mortality rate	Uncontrolled, before-after	NR	2	Structural bundle: 3 Equipment bundle: 5
Gilbert^48^ (2014)	Brazil			Develop an educational package and evaluate its impact on a range of neonatal outcomes	ITS	III	1	6
Gill^39^ (2009)	The Philippines			Evaluate the effectiveness of a package of infection control interventions	Uncontrolled, before-after	III	1	4
Picheansathian^45^ (2008)	Thailand			Identify the impact of a promotion programme on hand hygiene practices and its effect on nosocomial infection rates	Uncontrolled, before-after	NR	1	6
Villegas^40^ (2014)	Costa Rica			Determine the BSI rate of a NICU Quantify the impact of preventive measures on the BSI rate	Uncontrolled, before-after	III	1	2

**Type 5 - Composite Bundles**								

	
Agarwal^25^ (2007)	India			Evaluate the impact of simple interventions on neonatal mortality	Uncontrolled, before-after	III	1	10
Ahmed^52^ (2017)	Pakistan			Report outbreak of *S. marcescens* Determine the cause and source of the outbreak Report interventions to control the outbreak	Retrospective, descriptive	III	1	7
Ávila^53^ (2011)	Cuba			Report outbreak of *S. marcescens* Determine cause of the outbreak Report interventions to control outbreak	Retrospective, descriptive	NR	1	7
Balla^21^ (2018)β	India			Reduce neonatal CLABSI rates by 25% in three months and to sustain this over the next nine months	Uncontrolled, before-after	III	2	Main bundle: 4 CL maintenance bundle: 4
Calil^43^ (2001)	Brazil			Evaluate the efficacy of measures to control colonisation and infection by multiresistant bacteria	Uncontrolled, before-after	III	1	3
Cavicchiolo^44^ (2016) ε	Mozambique			To assess the effectiveness of interventions in terms of reduction of the neonatal mortality rate	Uncontrolled, before-after	NR	1	Clinical bundle: 10
Cetin^61^ (2015)	Turkey			Report outbreak of *S. maltophilia* Determine the cause and source of the outbreak Determine risk factors for infection Report outbreak management	Retrospective, analytical (case-control)	III	1	5
Chakrabarti^24^ (2001)	India			Report outbreak of *P. anomala* Determine the cause, source, and modes of transmission of the outbreak	Retrospective, analytical (case-control)	III	1	4
Chen^26^ (2015)	China			Evaluate the efficacy of different measures in preventing ICI in preterm infants < 33 weeks	Uncontrolled, before-after	III	1	6
Grey^50^ (2012)	Guatemala			Report outbreak of*K. pneumoniae* Determine the cause, source, and modes of transmission of the outbreak Report measures to control outbreak	Retrospective, descriptive	III	1	4
Hosoglu^62^ (2012)	Turkey			Report outbreak of *A. baumanii* Identify risk factors for *A. baumanii* Report measures to control outbreak	Retrospective, analytical (case-control)	III	1	8
Huang^30^ (2019)	China			Evaluate the efficacy of a bundle intervention on health care-associated MRSA infection	Uncontrolled, before-after	III	1	6
Hussain^33^ (2020)γ	Pakistan			Design a CLABSI prevention package to decrease CLABSI rates	Uncontrolled, before-after	III	3	Main bundle: 5 CL insertion bundle: 7 Prevention of fungal infections bundle: 3
Irfan^55^ (2019)	Pakistan			Report outbreak of MRSA Report measures to control outbreak	Retrospective, descriptive	II	1	11
Kulali^41^ (2019)	Turkey			Evaluate the effectiveness of bundled applications in the prevention of UVC-associated bloodstream infections	Uncontrolled, before-after	III	1	7
Landre-Peigne^32^ (2011)	Senegal			Evaluate the impact of a programme on the incidence of nosocomial bloodstream infections, neonatal mortality rates, the prevalence of drug-resistant strains and antimicrobial use	Uncontrolled, before-after	II	1	4
Mais^42^ (2015)	Lebanon			Evaluate the impact of quality improvement bundles on CLABSI rates	Uncontrolled, before-after	III	1	3
Miranda-Novales^60^ (2003)	Mexico			Report outbreak of *S. marcescens* Describe typing results using rapid pulsed-field gel electrophoresis and infection control measures	Retrospective, descriptive	III	1	4
Moore^56^ (2005)	Egypt			Report outbreak of *K. pneumoniae* Determine the cause and source of the outbreak Determine effectiveness of control measures	Retrospective, descriptive Uncontrolled, before-after	III	1	3
Mshana^59^ (2011)	Tanzania			Report outbreak of a novel *Enterobacter* sp. Perform molecular subtyping	Retrospective, descriptive	NR	1	4
Mwananyanda^47^ (2019)	Zambia			Evaluate the impact of an infection prevention control bundle on hospital-associated BSI and mortality	ITS	III	1	5
Narayan^54^ (2009)	Fiji			Report outbreak of *E. aerogenes* Determine the cause, source, and mode of transmission of the outbreak	Retrospective, descriptive	III	1	10
Qi^31^ (2018)	China			Report outbreak of *C. parapsilosis sensu stricto* Determine the cause and source of the outbreak Report measures to control outbreak	Retrospective, descriptive	III	1	6
Rahim^46^ (2009)	Malaysia			Implement education-based interventions to contribute to a reduction in nosocomial infections	Uncontrolled, before-after	III	1	3
Rosenthal^23^ (2012)	Argentina, Colombia, India, Mexico, Morocco, Peru, Philippines, El Salvador, Tunisia, Turkey			Evaluate the impact of the INICC multidimensional infection control programme on the reduction of VAP	Uncontrolled, before-after	III	1	11
Rosenthal^36^ (2013)δ	El Salvador, Mexico, The Philippines, and Tunisia			Evaluate the impact of INICC multidimensional infection control programme to reduce CLABSI	Uncontrolled, before-after	III	1	Main bundle: 6
Zhou^29^ (2013)	China			Evaluate the effectiveness of an intervention programme in decreasing neonatal VAP rate, neonatal mortality and the prevalence of drug-resistant strains	Uncontrolled, before-after	III	1	8
Zhou^28^ (2015)	China			Characterise CLABSI in a Chinese NICU Evaluate the impact of a multifaceted EPIQ program on CLABSI reduction	Uncontrolled, before-after	III	1	5

α Low- and middle-income countries where the included studies were performed as per World Bank definitions (2020).

β *Balla et al*.^21^ contains four bundles in the following groups: two *primary prevention*, one *implementation*, and one *composite*.

γ Hussain *et al*.^33^ contains four bundles in the following groups: one *primary prevention* and three *composite*.

δ Rosenthal *et al*.^36^ contains two bundles in the following groups: one *primary prevention* and one *composite*.

ε *Cavicchiolo et al*.^44^ contains three bundles in the following groups: two implementation and one composite. **Abbreviations**- BSI = bloodstream infections; CL = central line; CLABSI = central line-associated bloodstream infections; EENC = Early Essential Newborn Care; EPIQ = evidence-based practice for improving quality; ICI = invasive candida infections; INICC = International Nosocomial Infection Control Consortium; INCU = inpatient neonatal care units; ITS = interrupted time series; LOS = late-onset sepsis; MRSA = methicillin-resistant S. aureus; NICU = neonatal intensive care unit; NR = not reported; PICC = peripherally inserted central catheter; UVC = umbilical venous catheter; VAP = ventilator-associated pneumonia; VLBW = very low birth weight.

The studies reported data from 55 LMIC, with India and China being most commonly represented (Table 1).[20], 21, 22, 23, 24, 25, 26, 27, 28, 29, 30, 31 Regarding study design, 24 (55%) were uncontrolled before-after,^20^^,^^21^^,^^23^^,^^25^, ^26^, ^27^, ^28^, ^29^, ^30^^,^^32^, [33], 34, 35, 36, 37, 38, 39, 40, 41, 36, 43, 44, 45, 46 and two (5%) were interrupted time series.^47^^,^^48^ The rest were retrospective outbreak investigation studies.^12^^,^^22^^,^^24^^,^^31^^,^^49^, ^50^, ^51^, ^52^, ^53^, ^54^, ^55^, ^56^, ^57^, ^58^, ^59^, ^60^, ^61^, ^62^ 36 (82%) and two (5%) studies were performed in tertiary or secondary inpatient neonatal care units, respectively. Six (14%) studies did not report their care level setting (Table 1).^44^^,^^45^^,^^51^^,^^53^^,^^58^^,^^59^

A total of 56 care bundles were identified (Table 1). Three studies contained various care bundles inside another main care bundle.^21^^,^^33^^,^^36^ Three other studies described more than one bundle.^20^^,^^27^^,^^44^ The rest of the studies (39, 89%) described one bundle. The most common terms used in the publications to name the care bundles were ‘measures’ (13, 30%),^24^^,^^26^^,^^31^^,^^40^^,^^43^^,^^49^^,^^50^^,^^53^^,^^55^^,^^57^, ^58^, ^59^^,^^61^ followed by ‘bundles’ (nine, 21%).^20^^,^^23^^,^^27^^,^^30^^,^^34^^,^^35^^,^^37^^,^^41^^,^^47^ (Figure 2).

### The 3 + I Classification Framework for care bundle elements

#### Qualitative content analysis to construct the Classification Framework

The four main element groups of the 3 + I Classification Framework were created according to the four main themes (coding headings) identified: (1) *Primary prevention*, (2) *Detection* (i.e., secondary prevention elements), (3) *Case management* (i.e., tertiary prevention elements), and *Implementation* (3 + I*)*. A description of the main element themes and subthemes identified is represented in Table 2. A summary of the 3 + I Classification Framework is shown in Table 3 (for the complete taxonomy, see Table 3 in Appendix).

**Table 3:** Frequency of the 295 bundle elements according to the 3 + I Classification Framework.

Element classification				Bundle classification					Total number of elements per row (%)β
				Type 1 – Primary Prevention	Type 2 -Detectionα	Type 3 - Case Management	Type 4 -Implementation	Type 5 - Composite	
**Primary Prevention**									
1. Neonate	1.1. Feeding			1	··	··	··	1	2 (1·6)
	1.2. Skin-to-skin contact			1	··	··	··	··	1 (0·8)
	1.3. Skin disinfection			4	··	··	··	3	7 (5·5)
	1.4. Drug prescription			1	··	··	··	6	7 (5·5)
	1.5. Isolation			··	··	··	··	1	1 (0·8)
	1.6. Reduction of handling			··	··	··	··	1	1 (0·8)
2. Staff	2.1. HH			10	··	··	··	8	18 (14·1)
	2.2. Use of protocols/policies			1	··	··	··	1	2 (1·6)
	2.3. Organisation			··	··	··	··	2	2 (1·6)
	2.4. Contact barrier precautions			4	··	··	··	2	6 (4·7)
3. Caretaker	3.1. Empower mothers in routine care			··	··	··	··	1	1 (0·8)
4. Environment	4.1. Areas & equipment disinfection			··	··	··	··	3	3 (2·3)
	4.2. Waste disposal			··	··	··	··	1	1 (0·8)
	4.3. General unit organisation			1	··	··	··	4	5 (3·9)
5. Deviceγ	5.1. Catheter			36	··	··	··	19	55 (42·9)
	5.2. Ventilator			5	··	··	··	11	16 (12·5)
**Total number of primary prevention elements**				**64**	**··**	**··**	**··**	**64**	**128 (100)**

**Detection (Secondary Prevention)**									

1. Screening	1.1. New screening programme			··	··	··	··	2	2 (16·7)
2. Epidemiological surveillance	2.1. Implementation of new infection surveillance programme			··	··	··	··	3	3 (25·0)
	2.2. Enhance existing surveillance programmes			··	··	··	··	7	7 (58·3)
**Total number of detection elements**				**··**	**··**	**··**	**··**	**12**	**12 (100)**

**Case Management (Tertiary pPrevention)**									

1. Antibiotic prescription	1.1. Antibiotic policy & stewardship			··	··	1	··	8	9 (12·0)
2. Outbreak control	2.1. Neonate		2.1.1 Skin disinfection	··	··	1	··	1	2 (2·7)
			2.1.2 Feeding	··	··	1	··	··	1 (1·3)
			2.1.3 Drug prescription	··	··	1	··	··	1 (1·3)
			2.1.4 Isolation	··	··	2	··	3	5 (6·7)
	2.2. Staff		2.2.1. HH	··	··	4	··	4	8 (10·7)
			2.2.2. Use of protocols/policies	··	··	··	··	4	4 (5·3)
			2.2.3. Organisation	··	··	··	··	1	1 (1·3)
			2.2.4. Contact precautions	··	··	3	··	4	7 (9·3)
			2.2.5. Treatment of staff	··	··	··	··	1	1 (1·3)
	2.3. Environment		2.3.1. Areas & equipment disinfection	··	··	4	··	7	11 (14·7)
			2.3.2. General unit organisation	··	··	7	··	17	24 (32·0)
	2.4. Device	2.4.1. Catheter		··	··	··	··	1	1 (1·3)
**Total number of case management elements**				**··**	**··**	**24**	**··**	**51**	**75 (100)**

**Implementation**									

1. Audit & feedback				··	··	··	4	5	9 (11·3)
2. Change physical structure & equipment				··	··	··	12	8	20 (25·0)
3. Conduct educational meetings				··	··	··	10	25	35 (43·8)
4. Create/change credentialing and/or licensure standards				··	··	··	2	3	5 (6·3)
5. Create new clinical teams				··	··	··	··	1	1 (1·3)
6. Develop educational materials				··	··	··	··	3	3 (3·8)
7. Organise clinician implementation team meetings				··	··	··	··	1	1 (1·3)
8. Recruit, designate & train for leadership				··	··	··	··	1	1 (1·3)
9. Remind clinicians				··	··	··	1	1	2 (2·5)
10. Revise professional roles				··	··	··	··	3	3 (3·8)
**Total of implementation elements**				··	··	··	**29**	**51**	**80 (100)**

**Total number of elements per bundle group**				**64**	**··**	**24**	**29**	**178**	**295 (100)**δ

**Legend**: ^α^ No *detection* bundles in the studies. ^β^ Percentages are calculated using the total number of elements for each group as the denominator. ^γ^ Two devices were the target of all the bundle elements found in the literature: central line catheters and mechanical ventilators. No bundle elements were found for other medical devices. ^δ^ 305 bundle elements were identified in the included studies. However, only 295 of them were coded into the 3 + I Classification Framework because two elements could not be categorised due to a lack of detail to interpret their meaning and eight other elements were implemented exclusively in labour wards and not in neonatal wards (and therefore excluded). (··) is a zero value.

**Abbreviations**: HH = hand hygiene.

The *primary prevention* group was divided into five subgroups: *neonate, staff, caretaker, environment*, and *device* elements (Table 3). Two subgroups were found in the *detection* group: *screening* and *epidemiological surveillance*. Two subgroup themes were recognised in the *case management* group: *antibiotic prescription* and *outbreak control* initiatives. The *outbreak control* initiatives were further categorised using the same labels as those used in the *primary prevention* subgroups. Lastly, the *implementation* bundle elements found were grouped using ten of the 73 strategies compiled in the ERIC project (Table 4 in Appendix).^19^

After the bundle elements were categorised into the groups of the 3 + I Classification Framework, whole bundles were classified in different types according to the four element groups identified with an additional *composite* type for bundles containing a mixture of elements. Hence, there are a total of five bundle types: *primary prevention* (i.e. Type 1), *detection* (i.e. Type 2), *case management* (i.e. Type 3), *implementation* (i.e. Type 4), and *composite* (i.e. Type 5) (Table 3).

#### Quantitative analysis of the 3 + I Classification Framework

**Figure 3:**
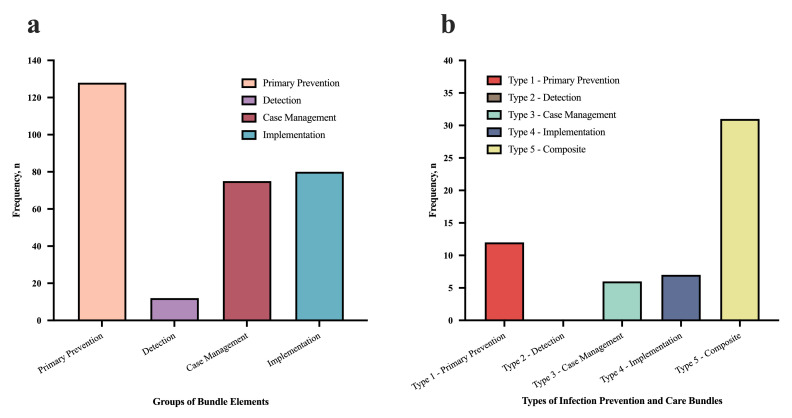
Frequency of the groups of bundle elements (3a) and frequency of the types of infection prevention and care bundles (3b). Colour legend: Figure 3a: Salmon = primary prevention; Violet = detection; Burgundy = case management; Blue = implementation. Figure 3b: Red = Type 1 – primary prevention; Grey = Type 2 – detection; Green = Type 3 – case management; Dark blue = Type 4 – implementation; Yellow = Type 5 – composite. After the creation of the four groups of the 3 + I Classification Framework using the bundle elements (i.e., prevention, detection, case management and implementation, represented in Figure 3a with their frequencies), whole bundles were also categorised into groups, according to the types of elements each one was made of (e.g., if one bundle contained four primary prevention elements, the whole bundle was categorised in primary prevention -Type 1- bundles. If one bundle contained a mixture of prevention, detection, case management, or implementation elements then the whole bundle was categorised into the composite -Type 5- group bundle). These are represented in Figure 3b.

Across all the extracted publications, 305 bundle elements were identified. 295 elements were coded into the 3 + I Classification Framework (Table 3). Two elements from two bundles (*"Control of risk factors"* and *"Taking meticulous care during invasive procedures"*) could not be categorised due to lack of detail to interpret their meaning.^26^^,^^49^ Two other bundles contained four bundle elements each implemented exclusively in labour wards, which were also excluded.^38^^,^^44^ Overall, the most common bundle elements detected were in the *primary prevention* group (128, 43%). 71(55%) of these *primary prevention* elements focused on advanced devices (i.e., central line catheters or mechanical ventilators). The *detection* element group had the least number of bundle elements within it (12, 4%). (Figure 3a).

12 (21%) of the bundles identified were classified as Type 1 bundles, six (11%) were Type 3 bundles, seven (13%) were Type 4 bundles, and 31 (55%) were Type 5. There were no bundles categorised as Type 2 (*detection*) (Figure 3b).

##### Primary prevention elements

A total of 128 (43%) elements were categorised as *primary prevention* (Table 3). The most frequent were *device* interventions (71, 55%), focused on central line catheters (55, 43%),^20^^,^^21^^,^^27^^,^^28^^,^[33], 34, 35, 36^,^^41^^,^^42^ and mechanical ventilators.^23^^,^^29^^,^^37^ No other elements directed to other devices were detected. 18 (14%) of the *primary prevention* elements were aimed at improving staff's hand hygiene. Only one element was found to promote breastfeeding,^38^ kangaroo mother care,^38^ or improve staff to patient ratios.^44^ 64 (50%) of the *primary prevention* elements identified were contained in the Type 1 bundles (*primary prevention*).

##### Detection (secondary prevention) elements

A total of 12 (4%) elements were aimed at detection (Table 3). The most frequent interventions were to enhance existing surveillance programmes (7, 58%)^43^^,^^52^^,^^53^^,^^59^^,^^60^ and to implement new ones (3, 25%).^36^^,^^62^^,^^63^ Two elements were focused on introducing new screening programmes.^30^^,^^46^ No bundles were exclusively made up of *detection* elements. Subsequently, bundles containing some *detection* elements were allocated to Type 5 (*composite*).

##### Case management (tertiary prevention) elements

75 (25%) elements were categorised under *case management* (Table 3). These were focused on *outbreak control* (66, 88%) or on improving *antibiotic policy and stewardship* in the neonatal units (9, 12%). In the *outbreak control* subgroup, 35 (47%) elements were aimed at the *environment* of the neonatal units, such as disinfection of areas and equipment or improving the organisation of the unit (e.g., use of a temporary ward, cohorting, or overcrowding reduction) (Table 3 in Appendix).^12^^,^^22^^,^^24^^,^^30^^,^^31^^,^^49^, ^50^, ^51^, ^52^, ^53^, ^54^, ^55^, ^56^, ^57^^,^^59^, ^60^, ^61^, ^62^ Additionally, 21 (28%) elements targeted the unit *staff* such as improving their hand hygiene or their use of protocols and policies. 9 (12%) elements were directed to the *neonate* such as their isolation, improving neonatal nutrition or local skin disinfection for venipuncture.^22^^,^^30^^,^^49^^,^^50^^,^^53^^,^^55^^,^^57^^,^^58^ There were no infection prevention and care bundles directly focused on the management and treatment of neonatal HCAI (e.g. no sepsis bundles) (not shown).

##### Implementation elements

80 (27%) of the elements identified being part of infection prevention and care bundles were implementation strategies (Table 3). The most frequently used was *conduct educational meetings* (35, 44%). 20 (25%) of the *implementation* elements found were aimed to *change physical structure & equipment* of the neonatal units including the provision of new and basic equipment and drug supplies to control outbreaks (e.g., provision of small medication bottles, single use fluid vials, or alcohol-based hand rub) (Table 3 in Appendix).^29^^,^^31^^,^^39^^,^^44^^,^^45^^,^^47^^,^^53^^,^^54^^,^^56^^,^^61^

## Discussion

This is the first scoping review to synthesise the published literature on infection prevention and care bundles addressing neonatal HCAI adverse outcomes in LMIC. 44 papers were found reporting 56 care bundles and categorising 295 elements. The 3 + I Classification Framework created in this study was useful in synthesising these into four mechanistic pathways. The majority of the elements were *primary prevention* interventions mostly focused on central line catheters and mechanical ventilators. There was a paucity of bundles and elements aimed at HCAI *detection* (4% of 295 elements). In addition, *case management* elements focused on the supportive care of neonates with HCAI were scarce. Although the United Nations targets the improvement of primary and secondary health care units for small and sick newborns to survive and thrive,^3^ only two (5%) of the studies were performed in secondary neonatal care units.

The absence of *detection* bundles and the fact that only a small number of *detection* elements were identified in the literature could support the evidence of a wide gap for HCAI detection and surveillance systems in LMIC hospitals reported in other studies.^6^^,^^64^ In LMIC, access to and quality of laboratory services are inconsistent and resource-constrained.^65^^,^^66^ Microbiological investigations such as blood cultures (a specimen sample prioritised by the World Health Organisation to launch routine epidemiological surveillance) are not usually performed as standard care.^67^^,^^68^^,^^69^ Health system factors that exacerbate this gap may include understaffing, uncovered training needs, and barriers to strengthening laboratory infrastructure and supply chains.^70^ Epidemiological detection and surveillance interventions are major milestones towards the reduction of HCAI attributed mortality and should be regarded as essential elements within the design of future care bundles.^66^ This gap is an opportunity to optimise the resources available and foster implementation research in the hospital services involved in HCAI detection and surveillance.^71^ For instance, research and development efforts focused on rapidpoint-of-care testing that can be performed in neonatal units could address these challenges in settings with limited laboratory capacity.^72^

Importantly, reported bundles in the literature consist mainly of *primary prevention* elements. However, these tended to focus on central line and mechanical ventilator devices which are exclusively used in neonatal intensive care units. No elements were orientated towards medical devices that reduce the most common causes of neonatal deaths in the levels of health care where most small and sick newborns are managed in LMIC (e.g., self-inflating bags, suctioning equipment, or incubators).^3^ These devices are also critical potential vectors of transmission of HCAI.^73^ Surprisingly, there was little or no mention of *neonate* subgroup elements in the *primary prevention* group such as breastfeeding or kangaroo mother care. These elements are simple, low-cost, evidence-based interventions that are proven to reduce HCAI in LMIC and are recommended by the World Health Organisation for infant care.^74^, ^75^, ^76^, ^77^ These elements can be easily introduced in all neonatal unit levels and income settings and could be the focus of future research efforts on bundles targeting HCAI *primary prevention*.

There were no *case management* bundles directly focused on the management and treatment of neonatal HCAI (such as sepsis bundles), and only a small number contained elements that addressed this. Possible reasons for this gap could be that they are included in the management and treatment guidelines or protocols as single interventions (which were outside of the scope of this review), or these might have been already established in the neonatal wards when the care bundles of the studies were designed.

The *implementation* elements aimed at *changing physical structure & equipment* are evidence that neonatal units in LMIC still lack access to essential equipment to prevent HCAI (e.g., availability of soap, sinks or smaller volume medication and fluid supplies to allow for their disposal in less than 24 h and avoid their reuse), as reported in other studies.^11^ These basic supplies need to be guaranteed in hospital settings to provide a safe care environment before scaling up the level of health care in these units. In addition, *implementation* elements are present in many of the identified bundles, reflecting that there is crossover in the reporting of bundled interventions and the techniques used to strengthen their implementation. This has also been noted in bundles used in adult health care.^78^ Although some implementation strategies can be considered as bundle elements (e.g. *conducting educational meetings*), a separate implementation reporting system would help to delineate the two, so that interventions may be reliably replicated.^79^

Most bundles were implemented in tertiary care neonatal units that provide resource-intensive support to a highly selected, small, and sick infant population. Similar to other reviews of studies in LMIC, we found secondary level units to be underrepresented.^80^^,^^81^ While the risk of HCAI in small and sick newborns is higher compared to term and sick neonates, there is under appreciation of the threat of HCAI in the rapidly expanding primary and secondary level care provision for neonates in LMIC.

This review presents evidence from a comprehensive search in five electronic databases and incorporated a snowball technique to identify other possible publications for inclusion. However, this review has limitations. First, the inclusion of clinical guidelines, management protocols and conference abstracts in the eligibility criteria could have yielded more infection prevention and care bundles. Second, laboratory settings were not included in the search strategy together with neonatal units. This may have reduced the identification of Type 2 (*detection*) bundles and more *detection* elements implemented in hospital laboratory services and therefore narrowed the holistic approach of the review. Nevertheless, key steps in the detection and surveillance pathways also occur in neonatal units, such as the performance of microbiologic tests or collection of clinical evidence from patients’ charts. To obtain an equilibrium between the breadth of the scoping review, its feasibility, and the time available for its completion, these were excluded from the search strategy. This balancing challenge is also acknowledged in other scoping reviews.^82^^,^^83^ Third, despite 100% of the full texts were independently reviewed by two authors in the second screening stage, only a random sample of 20 abstracts were double screened independently in the first stage. Although there was 100% agreement on this screening stage, there may have been residual selection bias. Fourth, 15 different terms were used to refer to care bundles in the included studies, demonstrating the need for consistent terminology to describe these types of interventions. In addition, the definition of a care bundle proposed by the Institute for Healthcare Improvement is very broad.^7^ This increased the difficulty in extracting well-defined bundle elements. To mitigate information bias, two reviewers conducted the bundle element extraction process. Despite this strategy, both reviewers agreed on their identification on 73% of studies. Finally, although the preparation stage in the qualitative analysis was supported by two researchers, only one of them performed the organisation stage to construct the Classification Framework. This could have been a source for misclassification bias.

The purpose of this review was to identify the range of potential bundles and bundle elements published in the literature to reduce HCAI and not to capture single interventions. Therefore, our individual findings for the four element groups identified (i.e., *primary prevention, detection, case management* and *implementation*) may not be generalisable beyond published studies on infection prevention and care bundles. Nevertheless, the 3 + I Classification Framework could help other researchers or health practitioners from LMIC in the design and evaluation of infection prevention and care bundles, regardless of the health care level. The elements can serve as potential “ingredients” for the construction of future multifaceted, holistic infection prevention and care bundles.

HCAI are a major threat to neonatal survival in LMIC and in urgent need of more evidence-based strategies for their reduction, especially to address the epidemic of antimicrobial resistance. Infection prevention and care bundles may be a promising approach contributing to the reduction of these infections. Using a scoping review methodology, this review synthesises the published literature on infection prevention and care bundles for inpatient newborn care units in LMIC. Our novel 3 + I Classification Framework for the care bundle elements could provide a useful basis for designing subsequent care bundles in all hospital settings for health care practice. Future research efforts should be directed towards the inclusion of infection *detection* elements in infection prevention and care bundles, particularly focused on point-of-care testing and surveillance. This target will be critical in resource-limited settings, where detection and surveillance systems are inadequate, yet the burden of infection is highest.

## Declaration of interests

We declare no competing interests.

## Appendix Supplementary materials

Image, application 1
