# Supplementary material for: Infection prevention and care bundles addressing health care-associated infections in neonatal care in low-middle income countries: a scoping review
Source: eClinicalMedicine. 2022 Jan 10;44:101259. doi: 10.1016/j.eclinm.2021.101259 (PMC8760419; doi:10.1016/j.eclinm.2021.101259)
Supplement: Supplementary file 1 [file mmc1.docx]

**Appendix - Supplementary material**

**Title**

Infection prevention and care bundles addressing health care-associated infections in neonatal care in low- middle income countries: a scoping review

**Authors**

Alexandra Molina García, James H. Cross, Elizabeth J. A. Fitchett, Kondwani Kawaza, Uduak Okomo, Naomi

E. Spotswood, Msandeni Chiume, Veronica Chinyere Ezeaka, Grace Irimu, Nahya Salim, Elizabeth M. Molyneux, Joy E. Lawn.

**NEST360 Infection Prevention, Detection and Care Collaborative Group**

Christine Bohne^1^, Helen Brotherton^2^, Tedbabe Degefie Hailegebriel^3^, David M. Goldfarb^4^, Danica Kumara^5^, Kirsty Le Doare^6^, Sara Liaghati-Mobarhan^7^, Goldy Mazia^8^, Vikas Meka^9^, Sarah Murless^10^, Hilda Namakula Mirembe^11^, John Baptiste Nkuranga^12^, Eric Ohuma^13^, Kara M. Palamountain^14^, Rebecca Penzias^15^, Rebecca Richards-Kortum^16^, Grace Tahuna Soko^17^.

## Affiliations

^1^ Rice360 Institute for Global Health Technologies, Rice University, Texas, USA, and Ifakara Health Institute, Ifakara, Tanzania.

^2^ Centre for Maternal, Adolescent, Reproductive & Child Health (MARCH), London School of Hygiene & Tropical Medicine, London, UK.

^3^ Health Program Group, United Nations International Children's Emergency Fund (UNICEF), New York,

USA.

^4^ Department of Pathology and Laboratory Medicine, BC Children's and Women's Hospital and University of British Columbia, Vancouver, Canada.

^5^ 3rd Stone Design Inc., California, USA.

^6^ Medical Research Council/Uganda Virus Research Institute (MRC/UVRI) and LSHTM Uganda Research Unit, Entebbe, Uganda and Infection and Immunity, St. George's, University of London, London, UK.

^7^ Rice360 Institute for Global Health Technologies, Rice University, Texas, USA.

^8^ PATH, Seattle, USA, and Save the Children, Westport, USA.

^9^ 3rd Stone Design Inc., California, USA.

^10^ Centre for Maternal, Adolescent, Reproductive & Child Health (MARCH), London School of Hygiene & Tropical Medicine, London, UK.

^11^ Adara Development, Uganda.

^12^ King Faisal Hospital, Kigali, Uganda.

^13^ Centre for Maternal, Adolescent, Reproductive & Child Health (MARCH), London School of Hygiene & Tropical Medicine, London, UK.

^14^ Kellogg School of Management, Northwestern University, Illinois, USA.

^15^ Centre for Maternal, Adolescent, Reproductive & Child Health (MARCH), London School of Hygiene & Tropical Medicine, London, UK.

^16^ Rice360 Institute for Global Health Technologies, Rice University, Texas, USA.

^17^ Newborn Essential Solutions and Technologies (NEST360), and Kamuzu University of Health Sciences, University of Malawi, Blantyre, Malawi.

**Table of Contents**

[TABLE 1. SEARCH STRATEGY 4](#_TOC_250005)

[TABLE 2. ELIGIBILITY CRITERIA 9](#_TOC_250004)

[TABLE 3. COMPLETE 3 + I CLASSIFICATION FRAMEWORK OF CARE BUNDLE ELEMENTS 10](#_TOC_250003)

[TABLE 4. DEFINITIONS OF THE TEN IMPLEMENTATION STRATEGIES USED, PROPOSED BY THE EXPERT RECOMMENDATIONS FOR IMPLEMENTING CHANGE (ERIC) PROJECT 14](#_TOC_250002)

[FIGURE 1. STUDY PROTOCOL 15](#_TOC_250001)

[FIGURE 2. QUALITATIVE INDUCTIVE CONTENT ANALYSIS METHODOLOGY FOR THE CONSTRUCTION OF THE CLASSIFICATION FRAMEWORK FOR THE CARE BUNDLES ELEMENTS 16](#_TOC_250000)

# Table 1. Search strategy

| **#1 Database: EMBASE** | |
| --- | --- |
| **Search #** | **Terms** |
| **Population terms** | |
| 1 | exp infant/ |
| 2 | infant*.mp. |
| 3 | exp newborn/ |
| 4 | neonat*.mp. |
| 5 | newborn*.mp. |
| 6 | (baby or babies).mp. |
| 7 | or/1-6 |
| **Concept terms – Health care-associated infections** | |
| 8 | exp epidemic/ or exp disease transmission/ |
| 9 | exp cross infection/ |
| 10 | infection*.mp. |
| 11 | sepsis.mp. |
| 12 | septic*.mp. |
| 13 | bacteri?emia.mp. |
| 14 | fung?emia.mp. |
| 15 | exp ventilator associated pneumonia/ or exp catheter infection/ |
| 16 | (central venous catheter or ((central or catheter or device) adj4 infection*) or clabsi).mp. |
| 17 | (pneumonia* or ventilator-associated or ventilator-acquired or vap).mp. |
| 18 | nosocomial*.mp. |
| 19 | outbreak*.mp. |
| 20 | (hospital* adj3 infection*).mp. |
| 21 | (disease* adj3 control*).mp. |
| 22 | epidemic*.mp. |
| 23 | cluster*.mp. |
| 24 | or/8-23 |
| **Concept terms – Care bundles** | |
| 25 | exp care bundle/ |
| 26 | bundle*.mp. |
| 27 | ((set* or multifaceted* or tailored or complex or care) adj3 intervention*).mp. |
| 28 | package*.mp. |
| 29 | checklist*.mp. |
| 30 | (care adj3 pathway*).mp. |
| 31 | or/25-30 |
| **Context terms – LMIC** | |
| 32-322 | LMIC terms |
| 323 | or/32-322 [ALL LOW AND MIDDLE-INCOME COUNTRIES] |

| 324 | and/7,24,31,323 |
| --- | --- |
| 325 | limit 324 to ((English or Spanish or French) and yr=" 2001 -Current") |

| **#2 Database: Pubmed** | |
| --- | --- |
| **Search #** | **Terms** |
| **Population terms** | |
| 1 | (((((((infant[MeSH Terms]) OR infant, newborn[MeSH Terms]) OR infant*[Text Word]) OR newborn*[Text Word]) OR neonat*[Text Word]) OR baby[Text Word]) OR babies[Text Word])) |
| **Concept terms – Health care-associated infections** | |
| 2 | ((((((((((((((((((((((((cross infection[MeSH Terms]) OR disease outbreaks[MeSH Terms]) OR disease transmission, infectious[MeSH Terms]) OR infection*[Text Word]) OR sepsis[Text Word]) OR septic*[Text Word]) OR septic shock[Text Word]) OR (bacteremia[Text Word] OR bacteraemia[Text Word])) OR fungemia[Text Word]) OR fungaemia[Text Word]) OR nosocomial*[Text Word]) OR cluster*[Text Word]) OR outbreak*[Text Word]) OR epidemic*[Text Word]) OR "disease control"[Text Word]) OR catheter related infection[MeSH Terms]) OR pneumonia, ventilator associated[MeSH Terms]) OR "central venous catheter"[Text Word]) OR "catheter associated"[Text Word]) OR clabsi[Text Word]) OR pneumonia*[Text Word]) OR "ventilator associated"[Text Word]) OR "ventilator acquired"[Text Word]) OR vap)) |
| **Concept terms – Care bundles** | |
| 3 | (((((((((patient care bundles[MeSH Terms]) OR bundle*[Text Word]) OR "complex intervention*"[Text Word]) OR "set* of intervention*"[Text Word]) OR "multifaceted intervention*"[Text Word]) OR "care intervention*"[Text Word]) OR package*[Text Word]) OR checklist*[Text Word]) OR "care pathway*"[Text Word])) |
| **Context terms – LMIC** | |
| 4 | LMIC filter |
| 5 | ((((((((((infant[MeSH Terms]) OR infant, newborn[MeSH Terms]) OR infant*[Text Word]) OR newborn*[Text Word]) OR neonat*[Text Word]) OR baby[Text Word]) OR babies[Text Word])) AND ((((((((((((((((((((((((cross infection[MeSH Terms]) OR disease outbreaks[MeSH Terms]) OR disease transmission, infectious[MeSH Terms]) OR infection*[Text Word]) OR sepsis[Text Word]) OR septic*[Text Word]) OR septic shock[Text Word]) OR (bacteremia[Text Word] OR bacteraemia[Text Word])) OR fungemia[Text Word]) OR fungaemia[Text Word]) OR nosocomial*[Text Word]) OR cluster*[Text Word]) OR outbreak*[Text Word]) OR epidemic*[Text Word]) OR "disease control"[Text Word]) OR catheter related infection[MeSH Terms]) OR pneumonia, ventilator associated[MeSH Terms]) OR "central venous catheter"[Text Word]) OR "catheter associated"[Text Word]) OR clabsi[Text Word]) OR pneumonia*[Text Word]) OR "ventilator associated"[Text Word]) OR "ventilator acquired"[Text Word]) OR vap)) AND (((((((((patient care bundles[MeSH Terms]) OR bundle*[Text Word]) OR "complex intervention*"[Text Word]) OR "set* of intervention*"[Text Word]) OR "multifaceted intervention*"[Text Word]) OR "care intervention*"[Text Word]) OR package*[Text Word]) OR checklist*[Text Word]) OR "care pathway*"[Text Word])) AND LMIC filter AND 2001/01/01:2020/12/31[Date - Publication]) AND (("English"[Language] OR "French"[Language]) OR "Spanish"[Language]) |

| **#3 Database: CINAHL** | |
| --- | --- |
| **Search #** | **Terms** |
| **Population terms** | |
| S1 | (MH "Infant, Newborn, Diseases+") OR (MH "Infant, Newborn+") |
| S2 | infant* or neonat* or newborn* or baby or babies |
| S3 | S1 OR S2 |
| **Concept terms – Health care-associated infections** | |
| S4 | (MH "Cross Infection+") |
| S5 | (MH "Disease Transmission+") OR (MM "Disease Transmission, Professional-to-Patient") OR (MH "Disease Transmission, Horizontal+") |
| S6 | (MM "Disease Outbreaks") |

| S7 | (MH "Catheter-Related Infections+") OR (MM "Catheter-Related Bloodstream Infections") OR (MM "Urinary Tract Infections, Catheter-Related") |
| --- | --- |
| S8 | (MM "Pneumonia, Ventilator-Associated") |
| S9 | infection* or sepsis or septic* bacteri?emia or fung?emia or nosocomial* or outbreak* or epidemic* or cluster* or "central venous catheter" or "central associated" or "device-associated" or clabsi or pneumonia* or "ventilator- associated" or "ventilator-acquired" or vap or (hospital* N3 infection*) or (disease* N3 control*) |
| S10 | S4 OR S5 OR S6 OR S7 OR S8 OR S9 |
| **Concept terms – Care bundles** | |
| S11 | (MH "Continuity of Patient Care+") OR (MH "Patient Care+") |
| S12 | bundle* or package* or checklist* or (care W3 pathway*) or ((set* or multifaceted* or tailored or complex or care) W3 intervention*) |
| S13 | S11 OR S12 |
| **Context terms – LMIC** | |
| S14-S31 | LMIC terms |
| S32 | LMIC terms combined with OR |
| S33 | S3 AND S10 ANDS13 AND S32 |

| **#4 Database: Global Health** | |
| --- | --- |
| **Search #** | **Terms** |
| **Population terms** | |
| 1 | exp infants/ |
| 2 | infant*.mp. |
| 3 | exp neonates/ |
| 4 | neonat*.mp. |
| 5 | newborn*.mp. |
| 6 | (baby or babies).mp. |
| 7 | or/1-6 |
| **Concept terms – Health care-associated infections** | |
| 8 | exp disease transmission/ or outbreaks.sh. |
| 9 | Cross infection/ |
| 10 | infection*.mp. |
| 11 | sepsis.mp. |
| 12 | septic*.mp. |
| 13 | bacteri?emia.mp. |
| 14 | fung?emia.mp. |
| 15 | exp ventilator associated pneumonia/ or bloodstream infections/ |
| 16 | (central venous catheter or ((central or catheter or device) adj4 infection*) or clabsi).mp. |
| 17 | (pneumonia* or ventilator-associated or ventilator-acquired or vap).mp. |
| 18 | nosocomial*.mp. |
| 19 | outbreak*.mp. |

| 20 | (hospital* adj3 infection*).mp. |
| --- | --- |
| 21 | (disease* adj3 control*).mp. |
| 22 | epidemic*.mp. |
| 23 | cluster*.mp. |
| 24 | or/8-23 |
| **Concept terms – Care bundles** | |
| 25 | exp disease control/ |
| 26 | bundle*.mp. |
| 27 | ((set* or multifaceted* or tailored or complex or care) adj3 intervention*).mp. |
| 28 | package*.mp. |
| 29 | checklist*.mp. |
| 30 | (care adj3 pathway*).mp. |
| 31 | or/25-30 |
| **Context terms – LMIC** | |
| 32-322 | LMIC terms |
| 323 | or/32-322 [ALL LOW AND MIDDLE-INCOME COUNTRIES] |
| 324 | and/7,24,31,323 |
| 325 | limit 324 to ((english or spanish or french) and yr=" 2001 -Current") |

| **#5 Database: Web of Science** | | | | | | | | | | | | | |
| --- | --- | --- | --- | --- | --- | --- | --- | --- | --- | --- | --- | --- | --- |
| **Search #** | **Terms** | | | | | | | | | | | | |
| **Population terms** | | | | | | | | | | | | | |
| #1 | TS=(infant*) | | OR | | TS=(neonat*) | | OR | TS=(newborn*) | | | OR | TS=(baby) | |
|  | OR TS=( babies) | | | | | | | | | | | | |
| **Concept terms – Hospital-acquired infections** | | | | | | | | | | | | | |
| #2 | TS=(infection*) | | | OR | | TS=(sepsis) | OR | TS=(septic*) | | OR | | TS=(bacteri$emia) |  |
|  | OR TS=(fung$emia) OR TS=(nosocomial*) OR TS=(outbreak*) OR TS=(epidemic*) OR TS=(cluster*) OR TS=(clabsi) OR TS=("cross-infection") | | | | | | | | | | | | |
| #3 | **TOPIC:** | ("central venous catheter") *OR* **TOPIC:** | | | | | | | ((central or catheter | | | | |
|  | device) NEAR/4 infection*) *OR* **TOPIC:** (clabsi) *OR* **TOPIC:** (pneumonia*) OR **TOPIC:** ("ventilator-associated") *OR* **TOPIC:** ("ventilator-associated pneumonia") *OR* **TOPIC:** ("ventilator associated pneumonia") *OR*  TOPIC: ("ventilator-acquired") *OR* **TOPIC:** (vap) *OR* **TOPIC:** (hospital* NEAR/3  infection*) *OR* **TOPIC:** (disease* NEAR/3 control*) | | | | | | | | | | | | |
| #4 | #3 OR #4 | | | | | | | | | | | | |
| **Concept terms – Care bundles** | | | | | | | | | | | | | |
| #5 | **TOPIC:** | (bundle*) *OR* **TOPIC:** | | | | | (package*) *OR* **TOPIC:** | | | | | (checklist*) | |
|  | *OR* **TOPIC:** (care NEAR/3 pathway*) *OR* **TOPIC:** ((set* or multifaceted or tailored or complex or care) NEAR/3 intervention*) *OR* **TOPIC:** (patient  NEAR/3 bundle*) | | | | | | | | | | | | |
| **Context terms – LMIC** | | | | | | | | | | | | | |
| #6 | LMIC terms | | | | | | | | | | | | |

#1 AND #4 AND #5 AND #6

**Refined by: PUBLICATION YEARS:** (2020 OR 2016 OR 2013 OR 2006 OR 2019 OR 2015 OR 2012 OR

**LANGUAGES:** ( ENGLISH )

#7

2017 OR 2014 OR 2010 ) AND

# Table 2. Eligibility criteria

| **Classification criteria** | **Inclusion criteria** | **Exclusion criteria** |
| --- | --- | --- |
| **1. Population** | Neonates | **Criteria 1:** infants more than 28 days of life and adults. |
| **2. Concept** | 2. 1. Care bundle 2.1.1. Studies reporting on care | **Criteria 2:** studies not reporting on care |
|  | bundles. | bundles (e.g., single interventions, guidelines, or |
|  |  | protocols). |
|  | 2.1.2. Studies where the bundle | **Criteria 3**: bundles where their elements are not |
|  | elements are described. | described. |
|  | 2.1.3. Studies reporting care bundles | **Criteria 4**: bundles exclusively focused on |
|  | that contain at least two bundle | other types of care (e.g., maternal, antenatal, or |
|  | elements related to HCAI prevention, | delivery) or with less than two bundle elements |
|  | detection, control, or management | related to HCAI prevention or control. |
|  | after birth. |  |
|  | 2.2. Outcomes Studies reporting on at least one of | **Criteria 5**: studies reporting exclusively on |
|  | the following adverse outcomes: | other results (e.g., hand hygiene compliance, |
|  | measures of HCAI disease frequency | risk factors for infection). |
|  | or exposure effect or any neonatal |  |
|  | outcomes. |  |
| **3. Context** | 3.1. Hospital units Inpatient neonatal care units (all  levels of care). | **Criteria 6**: other wards (e.g., paediatric intensive care, labour or maternity units). |
|  |  | **Criteria 7**: studies with results of neonatal |
|  |  | wards mixed with other wards. |
|  | 3.2. Country Studies done in low-middle income | **Criteria 8**: studies with a focus on high-income |
|  | settings. | countries. |
| **4. Study design** | All study designs. | No exclusion criteria. |
| **5. Types of** | All research studies. | **Criteria 9**: study protocols, conference |
| **publications** |  | abstracts, editorials, reviews, opinion articles, or studies with full text not accessible. |
| **6. Language** | English, Spanish, French. | **Criteria 10**: studies published in languages |
|  |  | other than English, Spanish, or French. |
| **7. Publication date** | 2001-2020 | **Criteria 11**: studies published before 2001 or after July 2020. |

**Abbreviations-** HCAI = health care-associated infections.

# Table 3. Complete 3 + I Classification Framework of care bundle elements

| **Element Classification Bundle Classification** | | | | | | | | |
| --- | --- | --- | --- | --- | --- | --- | --- | --- |
|  |  |  | | **Type 1 -** | **Type 2 -** | **Type 3 -** | **Type 4 -** | **Type 5 -** |
|  |  |  | | **Primary** | **Detection^α^** | **Case** | **Implementation** | **Composite** |
|  |  |  | | **Prevention** |  | **Management** |  |  |
| **Primary Prevention** |  |  | |  |  |  |  |  |
| 1. Neonate | 1.1 Feeding | 1.1.1 Breastfeeding | | 1 | ·· | ·· | ·· | ·· |
|  |  | 1.1.2 Enteral feeding | | ·· | ·· | ·· | ·· | 1 |
|  | 1.2 Skin-to-skin contact/KMC |  | | 1 | ·· | ·· | ·· | ·· |
|  | 1.3 Skin disinfection | 1.3.1 Local sites | | 4 | ·· | ·· | ·· | 2 |
|  |  | 1.3.2 Complete CHX cleansing | | ·· | ·· | ·· | ·· | 1 |
|  | 1.4 Drug prescription |  | | ·· | ·· | ·· | ·· | 1 |
|  |  | 1.4.1 Fungal prophylaxis | | ·· | ·· | ·· | ·· | 4 |
|  |  | 1.4.2 Avoid use of risk factors  drugs for HCAI | | ·· | ·· | ·· | ·· | 1 |
|  |  | 1.4.3 Anticoagulant prophylaxis | | 1 | ·· | ·· | ·· | ·· |
|  | 1.5 Isolation |  | | ·· | ·· | ·· | ·· | 1 |
|  | 1.6 Reduction of handling |  | | ·· | ·· | ·· | ·· | 1 |
| 2. Staff | 2.1 HH |  | | 10 | ·· | ·· | ·· | 8 |
|  | 2.2 Use of protocols/policies |  | | 1 | ·· | ·· | ·· | 1 |
|  | 2.3 Organisation | 2.3.1 Staff to patient ratios | | ·· | ·· | ·· | ·· | 1 |
|  |  | 2.3.2 Staff shifts | | ·· | ·· | ·· | ·· | 1 |
|  | 2.4 Contact barrier precautions |  | | 4 | ·· | ·· | ·· | 2 |
| 3. Caretaker | 3.1 Empower mothers in routine |  | | ·· | ·· | ·· | ·· | 1 |
|  | care practices |  |  |  |  |  |  |  |
| 4. Environment | 4.1 Areas & equipment disinfection |  |  | ·· | ·· | ·· | ·· | 3 |
|  | 4.2 Waste disposal |  |  | ·· | ·· | ·· | ·· | 1 |
|  | 4.3 General unit organisation | 4.3.1 Early discharge |  | ·· | ·· | ·· | ·· | 2 |
|  |  | 4.3.2 Rational admissions |  | 1 | ·· | ·· | ·· | 1 |
|  |  | 4.3.3 Restriction of ward visits |  | ·· | ·· | ·· | ·· | 1 |
| 5. Device | 5.1 Catheter | 5.1.1 Central |  | ·· | ·· | ·· | ·· | 5 |
|  |  |  | 5.1.1.1 Aseptic technique during CL  manipulation/insertion | 8 | ·· | ·· | ·· | 1 |
|  |  |  | 5.1.1.2 Dressing management | 6 | ·· | ·· | ·· | 3 |
|  |  |  | 5.1.1.3 Evaluation of CL indication | 7 | ·· | ·· | ·· | 3 |
|  |  |  | 5.1.1.4 Evaluation of CL insertion | 2 | ·· | ·· | ·· | ·· |
| sites | | | |  |  |  |  |  |
| 5.1.1.5 Avoid femoral site | | | | 4 | ·· | ·· | ·· | ·· |
|  |  |  | 5.1.1.6 CL access systems | 6 | ·· | ·· | ·· | 4 |
| management & setup | | | |  |  |  |  |  |
| 5.1.1.7 Use CL carts/kits | | | | 3 | ·· | ·· | ·· | 2 |
|  |  |  | 5.1.1.8 Preferential access of CL | ·· | ·· | ·· | ·· | 1 |
|  | |  | during the day |  |  |  |  |  |
| 5.2 Ventilator | | 5.2.1 Evaluation of MV indication |  | 1 | ·· | ·· | ·· | 2 |
|  | | 5.2.2 Oral care |  | 1 | ·· | ·· | ·· | 1 |
|  | | 5.2.3 Weaning assessment |  | ·· | ·· | ·· | ·· | 1 |

- - 1. Preferential use of orotracheal intubation

·· ·· ·· ·· 1

- - 1. Avoid gastric distension ·· ·· ·· ·· 1
    2. Avoid medications that increase risk of VAP

| ·· | ·· | ·· | ·· | 1 |
| --- | --- | --- | --- | --- |
| 1 | ·· | ·· | ·· | 3 |

- - 1. Ventilator circuits

programmes

| management  5.2.8 Head of bed elevation 30-45º | 1 | ·· | ·· | ·· | ·· |
| --- | --- | --- | --- | --- | --- |
| 5.2.9 Sterile technique during ventilator manipulation | 1 | ·· | ·· | ·· | 1 |
| **Total number of primary prevention elements** | **64** | **··** | **·** | **··** | **64** |
| **Detection (secondary prevention)** |  |  |  |  |  |
| 1. Screening 1.1 New screening programme | ·· | ·· | ·· | ·· | 2 |
| 2. Epidemiological 2.1 Implementation of infection  Surveillance surveillance programmes | ·· | ·· | ·· | ·· | 3 |
| 2.2 Enhance existing surveillance | ·· | ·· | ·· | ·· | 4 |

- - 1. Neonatal risk factors for infection

| ·· | ·· | ·· | ·· | 1 |
| --- | --- | --- | --- | --- |
| ·· | ·· | ·· | ·· | 1 |

- - 1. Improve time for culture and

| disinfection |  | | | | | |
| --- | --- | --- | --- | --- | --- | --- |
| 2.3.2 General unit organisation | 2.3.2.1 Use of temporary ward | ·· | ·· | 1 | ·· | 1 |
|  | 2.3.2.2 Overcrowding reduction | ·· | ·· | 1 | ·· | 2 |
|  | 2.3.2.3 Unit doors locked | ·· | ·· | ·· | ·· | 1 |
|  | 2.3.2.4 Temporary unit closure | ·· | ·· | 1 | ·· | ·· |
|  | 2.3.2.5 Rational admissions | ·· | ·· | 1 | ·· | 3 |
|  | 2.3.2.6 Improve equipment use | ·· | ·· | 2 | ·· | 3 |
|  | 2.3.2.7 Cohorting | ·· | ·· | 1 | ·· | 7 |

|  | sensitivity results |  | | | | | |
| --- | --- | --- | --- | --- | --- | --- | --- |
|  | 2.2.3 Improve communication |  |  |  |  |  |  |
|  | between neonatologists and |  | ·· | ·· | ·· | ·· | 1 |
|  | microbiologist |  |  |  |  |  |  |
| **Total number of detection elements** |  | **··** | **··** | **··** | **··** | **12** |  |
| **Case Management (tertiary prevention)** |  |  |  |  |  |  |  |
| 1. Antibiotic prescription 1.1 Antibiotic policy & stewardship |  |  | ·· | ·· | 1 | ·· | 8 |
| 2. Outbreak control 2.1 Neonate | 2.1.1 Skin disinfection | 2.1.1.1 Local for venipuncture | ·· | ·· | 1 | ·· | 1 |
|  | 2.1.2 Feeding |  | ·· | ·· | 1 | ·· | ·· |
|  | 2.1.3 Drug prescription | 2.1.3.1 Avoid use of sedation | ·· | ·· | 1 | ·· | ·· |
|  | 2.1.4 Isolation |  | ·· | ·· | 2 | ·· | 3 |
| 2.2 Staff | 2.2.1 HH |  | ·· | ·· | 4 | ·· | 4 |
|  | 2.2.2 Use of protocols/policies |  | ·· | ·· | ·· | ·· | 4 |
|  | 2.2.3 Organisation | 2.2.3.1 Staff workload reduction | ·· | ·· | ·· | ·· | 1 |
|  | 2.2.4 Contact barrier precautions |  | ·· | ·· | ·· | ·· | 3 |
|  |  | 2.2.4.1 Gloves | ·· | ·· | 2 | ·· | 1 |
|  |  | 2.2.4.2 Face masks | ·· | ·· | 1 | ·· | ·· |
|  | 2.2.5 Treatment of staff carriers |  | ·· | ·· | ·· | ·· | 1 |
| 2.3 Environment | 2.3.1 Areas & equipment |  | ·· | ·· | 4 | ·· | 7 |

2.4 Device 2.4.1 Catheter 2.4.1.1 Aseptic technique during manipulation of IV line

·· ·· ·· ·· 1

| **Total number of case management elements** | **··** |  | **··** | **24** | **··** | **51** |
| --- | --- | --- | --- | --- | --- | --- |
| **Implementation** |  |  |  |  |  |  |
| 1. Audit & feedback |  | ·· | ·· | ·· | 4 | 5 |

1. Change physical structure & equipment

|  | | ·· | ·· | ·· | 3 | 1 |
| --- | --- | --- | --- | --- | --- | --- |
| 2.2 Drug supplies | 2.2.1 Antibiotics | ·· | ·· | ·· | 1 | ·· |
|  | 2.2.2 Small medication bottles | ·· | ·· | ·· | ·· | 2 |
|  | 2.2.3 Single-use fluid vials | ·· | ·· | ·· | ·· | 1 |
| 2.3 Staff equipment | 2.3.1 Alcohol-based hand rub | ·· | ·· | ·· | 3 | 2 |
|  | 2.3.2 Clothing | ·· | ·· | ·· | ·· | 1 |
|  | 2.3.3 Hand towels | ·· | ·· | ·· | 1 | ·· |
| 2.4 Environment equipment | 2.4.1 Disposable disinfectant wipes | ·· | ·· | ·· | ·· | 1 |
| 2.5 Device equipment | 2.5.1 Oxygen concentrators | ·· | ·· | ·· | 1 | ·· |
|  | 2.5.2 Phototherapy | ·· | ·· | ·· | 1 | ·· |
|  | 2.5.3 Infusion pumps | ·· | ·· | ·· | 1 | ·· |
|  | 2.5.4 Glucometer | ·· | ·· | ·· | 1 | ·· |
| 3.1 Neonate | 3.1.1 Essential newborn care | ·· | ·· | ·· | 2 | 2 |
|  | 3.1.2 Advanced newborn care | ·· | ·· | ·· | 3 | ·· |
| 3.2 Environment | 3.2.1 Areas & equipment cleaning | ·· | ·· | ·· | ·· | 2 |
| 3.3 Device | 3.3.1 CL | ·· | ·· | ·· | 2 | 4 |
|  | 3.3.2 MV | ·· | ·· | ·· | ·· | 1 |
|  | 3.3.3 VAP | ·· | ·· | ·· | ·· | 1 |
| 3.4 Infection prevention & control |  | ·· | ·· | ·· | 2 | 11 |
|  | 3.4.1 HH | ·· | ·· | ·· | 1 | 4 |
|  |  | ·· | ·· | ·· | 2 | 3 |
|  |  | ·· | ·· | ·· | ·· | 1 |
| 6.1 Management protocols |  | ·· | ·· | ·· | ·· | 2 |
| 6.2 Admission policy |  | ·· | ·· | ·· | ·· | 1 |
|  |  | ·· | ·· | ·· | ·· | 1 |
|  |  | ·· | ·· | ·· | ·· | 1 |
| 9.1 Text messages |  | ·· | ·· | ·· | ·· | 1 |
| 9.2 Posters |  | ·· | ·· | ·· | 1 | ·· |

1. Conduct educational meetings
2. Create/change credentialing and/or licensure standards
3. Create new clinical teams
4. Develop educational materials
5. Organise clinician implementation team meetings
6. Recruit, designate & train for leadership
7. Remind clinicians

2.1 Physical structure

| 10. Revise professional roles |  | ·· | ·· | ·· | ·· | 3 |
| --- | --- | --- | --- | --- | --- | --- |
| **Total of implementation elements** | **··** |  | **··** | **··** | **29** | **51** |
| **Total number of elements per bundle group (%)** |  | **64** | **··** | **24** | **29** | **178** |

**Legend-** a No *detection* bundles in the studies. (··) is a zero value.

**Abbreviations-** CHX = chlorhexidine; CL = central line; HCAI = health care-associated infections; HH = hand hygiene; IV: = intravenous KMC = kangaroo mother care; MRSA = methicillin-resistant S. aureus; MV = mechanical ventilation; VAP = ventilator-associated pneumonia.

# Table 4. Definitions of the ten implementation strategies used, proposed by the Expert Recommendations for Implementing Change (ERIC) Project

**Strategy Definitions**

**Audit and provide feedback** Collect and summarise clinical performance data over a specified time period and give it

to clinicians and administrators to monitor, evaluate, and modify provider behaviour

**Change physical structure and equipment**

Evaluate current configurations and adapt, as needed, the physical structure and/or equipment (e.g., changing the layout of a room, adding equipment) to best accommodate the targeted innovation

**Conduct educational meetings** Hold meetings targeted toward different stakeholder groups (e.g., providers,

administrators, other organisational stakeholders, and community, patient/consumer, and family stakeholders) to teach them about the clinical innovation

**Create new clinical teams** Change who serves on the clinical team, adding different disciplines and different skills

to make it more likely that the clinical innovation is delivered (or is more successfully delivered)

**Create or change credentialing and/or licensure standards**

Create an organisation that certifies clinicians in the innovation or encourage an existing organisation to do so. Change governmental, professional certification or licensure requirements to include delivering the innovation. Work to alter continuing education requirements to shape professional practice toward the innovation

**Develop educational materials** Develop and format manuals, toolkits, and other supporting materials in ways that make

it easier for stakeholders to learn about the innovation and for clinicians to learn how to deliver the clinical innovation

**Organise clinician implementation team meetings**

**Recruit, designate, and train for leadership**

Develop and support teams of clinicians who are implementing the innovation and give them protected time to reflect on the implementation effort, share lessons learned, and support one another's learning

Recruit, designate, and train leaders for the change effort

**Remind clinicians** Develop reminder systems designed to help clinicians to recall information and/or prompt them to use the clinical innovation

**Revise professional roles** Shift and revise roles among professionals who provide care and redesign job

characteristics

# Figure 1. Study protocol

**Infection prevention and care bundles addressing hospital-acquired infections in neonatal care in LMIC a scoping review protocol**

**Infection prevention and care bundles addressing hospital-acquired infections in neonatal care in low- middle income countries a scoping review protocol_Appendix**

**Study Protocol**

**Title**

**Infection prevention and care bundles addressing hospital-acquired infections in neonatal care in low- middle income countries: a scoping review protocol.**

**Authors**

Alexandra Molina Garcia^1^, James H Cross^1^, Elizabeth J A Fitchett^2^, Kondwani Kawaza^3^, Uduak Okomo^4^, Naomi Spotswood^5^, Msandeni Chiume^3^, Chinyere Ezeaka^6^, Grace Irimu^7^, Nahya Salim^8^, Elizabeth M Molyneux^3^, Joy E Lawn^1^.

**Affiliations**

^1^ MARCH Centre, London School of Hygiene & Tropical Medicine, London, UK.

^2^ UCL Great Ormond Street Institute of Child Health, London, UK.

^3^ Department of Paediatrics, College of Medicine, University of Malawi, Blantyre, Malawi.

^4^ Medical Research Council Unit The Gambia at London School of Hygiene & Tropical Medicine, Fajara, The Gambia.

^5^ The Macfarlane Burnet Institute for Medical Research and Public Health, Melbourne, Australia.

^6^ Department of Paediatrics, College of Medicine, University of Lagos, Nigeria.

^7^ Department of Paediatrics and Child Health, University of Nairobi, Kenya.

^8^ Department of Paediatrics and Child Health, Muhimbili University of Health and Allied Sciences, Dar Es Salaam, Tanzania.

**Introduction**

In 2019, 2,440,000 neonates died worldwide. Almost 80% of these deaths occurred in sub-Saharan Africa and Central and Southern Asia,^1^ of which infections were one of the leading causes, accounting for 23%.^2^ In 2015, the Sustainable Development Goals were set by the United Nations to reduce national neonatal deaths rates to 10 per 1,000 live births by 2035.^3^ However, the latest estimates of neonatal mortality rates show figures still as high as 27 deaths per 1,000 live births in sub-Saharan Africa.^1^ If this target is to be achieved, neonatal mortality reduction still needs to be considered a major public health concern.

The emergence of hospital-acquired infections (HAI) and associated antimicrobial resistance is a significant threat to reducing neonatal mortality.^4^ In inpatient neonatal care units in low- and middle- income countries (LMIC), HAI incidence is estimated to be 15.2 to 62.0 per 1,000 patient-days, nine-fold times higher than observed in some high-income settings.^5^ These striking figures may grow substantially as institutional deliveries have increased to almost 80% worldwide and neonatal care in health facilities is expanding in

developing countries.^6,7^ Infection prevention and control interventions need to be implemented into daily neonatal care to reduce HAI neonatal mortality and improve healthcare quality. These interventions must adopt a holistic approach, addressing the different sources (e.g. healthcare staff, the neonate, or ward equipment) and stages (e.g. prevention, detection, or control) of infection.

Care bundles are a new strategy developed by the Institute for Healthcare Improvement to improve the quality of care in adult intensive care units in 2001.^8^ They are a small group of evidence-based practices (called ‘elements’) implemented together to improve clinical outcomes. The use of care bundles has extended rapidy to neonatal settings with demonstrated success in reducing adverse clinical outcomes.^9-11^ Thus, a strong case exists to promote research on care bundles as a promising strategy to reduce the burden of HAI on neonatal mortality. As a result, there is a need to develop a care bundle classification system as a first step towards advancing in this area of research.

Existing systematic reviews on care bundles in neonatal settings focus on evaluating their effectiveness to reduce central line- or ventilator-associated infections.^9,10^ However, prior reviews have not assembled published literature on care bundles in inpatient care units addressing all types of HAI in LMIC regardless of study design or objectives, with no existing framework to categorise care bundles and their elements. This taxonomy could allow the grouping of bundle elements into similar interventions, establishing a conceptual framework for consistent terminology for care bundles and bundle element categories and their definitions. Additionally, these can be used as potential “ingredients” for the construction of future neonatal care bundles to reduce HAI in other LMIC hospital settings.

This scoping review aims to identify and synthesise published literature on infection prevention and care bundles addressing neonatal HAI in LMIC. Specific objectives were:

1. To develop a classification framework for the elements of care bundles (i.e. based on the bundle elements identified in the studies).
2. To describe the content of the classification framework developed.
3. To identify evidence gaps found within this taxonomy to target future research.

The most appropriate evidence synthesis methodology to answer the stated aims and objectives is a scoping review^12,13^. Scoping reviews are used to map the existing research using different study designs in a given field^14^. Using this approach, one can identify and examine the breadth of the published literature on care bundles and their elements addressing HAI in inpatient neonatal care units in LMIC.

To ensure rigor and transparency, this scoping review will be based on the guidance framework for conducting scoping reviews developed by the Joanna Briggs Institute (JBI)^12^. It will be reported using the Preferred Reporting Items for Systematic Reviews and Meta-Analyses Extension for Scoping Reviews (PRISMA ScR)^15^

**Inclusion criteria**

## Types of participants

The target population of this review are neonates (infants less than 28 days of life).

## Concept

Two concepts are determined for this scoping review. The first is care bundles (defined by the Institute for Healthcare Improvement as: "*A small set of evidence-based interventions for a defined patient segment/population and care setting that, when implemented together, will result in significantly better outcomes than when implemented individually*").^8^ Secondly, studies will also be included if at least one of the following outcomes were reported: HAI measures of disease frequency or exposure effect or any negative neonatal outcomes (ie. overall mortality, morbidity, death before discharge, or survival rates). HAI is defined according to the Center for Disease Control as a localised or systemic infection occurring at least 48 h after hospital admission or within one week of discharge with evidence of infection related to a previous hospitalisation^16^.

## Context

Studies must be set in inpatient care units in LMIC.

Studies will be included if the identified bundles contained at least two elements related to HAI prevention or control after birth implemented in inpatient care units. All study designs will be included.

Exclusion criteria are:

1. Studies set in other wards (e.g. paediatric intensive care, labour, maternity, or emergency units).
2. Studies with results including infants older than 28 days.
3. Studies reporting on guidelines, single interventions, management protocols, conference abstracts, editorials, reviews, study protocols, opinion articles, or publications were the full-text could not be accessed.

**Search strategy**

The literature search was performed across five electronic databases: EMBASE, Pubmed, Global Health, CINAHL, and Web of Science. The search strategy includes English keywords and medical subject headings for four concepts: neonates, care bundles, HAI, and LMIC (appendix − Table 1). To include only studies from LMIC, personalised country lists were created for CINHAL and Web of Science, and pre-established country lists were used for the rest of the databases, as per World Bank definitions. In 2001, the Institute for Healthcare

Improvement developed the concept of care bundles, therefore, searches will also be limited to studies published from 2001 until July 3rd, 2020 in only English, Spanish or French languages.

**Sources of evidence selection**

The records identified will be imported into the reference manager EndNote X9.3.3 (Clarivate Analytics, Boston, Massachusetts, USA) for the removal of duplicates. In a first stage, all studies will be screened by one reviewer (AMG) by title and abstract. A second reviewer (EJAF) will screen a random sample of 20 of these studies. In a second stage, all full-text articles will be assessed for eligibility independently by both reviewers. Any disagreements in this stage will be resolved by consensus.

**Data extraction**

The studies included in the review will be exported to Microsoft Excel 16.40 (Microsoft, Redmond, Washington, USA) for the data charting process. This chart will be piloted by AMG and EJAF on two articles. The following data will be extracted by AMG:

Study characteristics: first author, year of publication, aim, country of origin, study design, level of inpatient care unit, terminology found in the methods section of the articles to describe the care bundles (if unavailable, in the title and abstract).

Bundle elements: number and description of elements in the bundle (this information will be extracted by both reviewers. If disagreements were also observed in this stage, these will be resolved by consensus).

Study designs were classified using the algorithm proposed by the National Institute for Health and Care Excellence (NICE) (appendix − Figure 1). Quasi-experimental studies were categorised according to the definitions proposed by the Cochrane Effective Practice and Organisation of Care (EPOC) (appendix − Figure 2).

**Analysis of the evidence and methodology to create the Classification Framework**

The synthesis will be displayed narratively, including quantitative and qualitative analysis. Quantitative analysis (frequencies and percentages) will be performed to report the study characteristics. Qualitative inductive content analysis will be carried out to build the classification framework for the bundle elements. This has been used in previous published scoping reviews^17,18^. An inductive approach will be used as no previous care bundle element framework was identified in the literature. This analysis follows the three-step process proposed by Elo and colleagues: preparation, organisation and reporting.^19^ In the preparation step, bundle elements will be extracted from the articles and read numerous times. In the organisation step, each bundle elements will be coded with a heading chosen according to their meaning. Following this, bundle element headings will be grouped into higher order headings with shared meanings using an iterative process to create the main themes (groups) and subthemes (subgroups). Names and definitions will be provided to the main

themes and subthemes. If implementation themes are identified, these will be named after the categories of the Expert Recommendations for Implementing Change (ERIC) study.^20^

To further synthesise the results, once the bundle elements are coded and categorised, whole bundles will also be categorised. In the reporting step, a description and quantitative analysis (frequencies and percentages) of the content of the categories of the bundle element classification framework will be performed. Based on scoping review methodological guidelines,^12^ critical appraisal of the quality of the included studies will not be described.

**Presentation of results**

A word cloud will be generated using Wordle (Jonathan Feinberg, 2014, http://www.wordle.net). GraphPad Prism version 8 (GraphPad software, San Diego, California, USA) software will be used to produce the graphical presentation.

**References**

1. United Nations Inter-agency Group for Child Mortality Estimation (UN IGME). Levels & Trends in Child Mortality: Report 2020, Estimates developed by the United Nations Inter-agency Group for Child Mortality

Estimation. New York: United Nations Children’s Fund; 2020.

1. United Nations Inter-agency Group for Child Mortality Estimation (UN IGME). Levels & Trends in Child Mortality: Report 2019, Estimates developed by the United Nations Inter-agency Group for Child Mortality Estimation. New York; 2019.
2. Lawn JE, Blencowe H, Oza S, et al. Every Newborn: progress, priorities, and potential beyond survival.

*Lancet* 2014; **384 North American Edition**(9938): 189-205.

1. Laxminarayan R, Bhutta Z. Antimicrobial resistance—a threat to neonate survival. *The Lancet Global Health* 2016; **4**(10): e676-e7.
2. Allegranzi B, Nejad SB, Combescure C, et al. Burden of endemic health-care-associated infection in developing countries: systematic review and meta-analysis. *The Lancet* 2011; **377**(9761): 228-41.
3. United Nations International Children’s Emergency Fund (UNICEF). Delivery care. 2020. https://data.unicef.org/topic/maternal-health/delivery-care/ (accessed 22 Feb 2021).
4. World Health Organisation. Survive and thrive: transforming care for every small and sick Newborn. Geneva: World Health Organisation, 2019.
5. Resar R, Griffin FA, Haraden C, Nolan TW. Using Care Bundles to Improve Health Care Quality. Cambridge, Massachusetts: Institute for Healthcare Improvement 2012.
6. Payne V, Hall M, Prieto J, Johnson M. Care bundles to reduce central line-associated bloodstream infections in the neonatal unit: A systematic review and meta-analysis. *Archives of Disease in Childhood: Fetal and Neonatal Edition* 2018; **103**(5): F422-F9.
7. Niedzwiecka T, Patton D, Walsh S, Moore Z, O'Connor T, Nugent L. What are the effects of care bundles on the incidence of ventilator-associated pneumonia in paediatric and neonatal intensive care units? A systematic review. *J Spec Pediatr Nurs* 2019; **24**(4): e12264.
8. Schlapbach LJ, Javouhey E, Jansen NJG. Paediatric sepsis: old wine in new bottles? *Intensive Care Med*

2017; **43**(11): 1686-9.

1. Aromataris E, Munn Z. JBI Reviewer’s Manual: Joanna Briggs Institute; 2020.
2. Munn Z, Peters MDJ, Stern C, Tufanaru C, McArthur A, Aromataris E. Systematic review or scoping review? Guidance for authors when choosing between a systematic or scoping review approach. *BMC Med Res Methodol* 2018; **18**(1): 143.
3. Arksey H, O'Malley L. Scoping studies: towards a methodological framework. *International Journal of Social Research Methodology: Theory & Practice* 2005; **8**: 19-32.
4. Tricco AC, Lillie E, Zarin W, et al. PRISMA Extension for Scoping Reviews (PRISMA-ScR): Checklist and Explanation. *Annals of Internal Medicine* 2018; **169**(7).
5. Horan TC, Andrus M, Dudeck MA. CDC/NHSN surveillance definition of health care-associated infection and criteria for specific types of infections in the acute care setting. *Am J Infect Control* 2008; **36**(5): 309-32.
6. Harfield SG, Davy C, McArthur A, Munn Z, Brown A, Brown N. Characteristics of Indigenous primary health care service delivery models: a systematic scoping review. *Global Health* 2018; **14**(1): 12.
7. Olding M, McMillan SE, Reeves S, Schmitt MH, Puntillo K, Kitto S. Patient and family involvement in adult critical and intensive care settings: a scoping review. *Health Expect* 2016; **19**(6): 1183-202.
8. Elo S, Kyngas H. The qualitative content analysis process. *J Adv Nurs* 2008; **62**(1): 107-15.
9. Powell BJ, Waltz TJ, Chinman MJ, et al. A refined compilation of implementation strategies: results from the Expert Recommendations for Implementing Change (ERIC) project. *Implement Sci* 2015; **10**: 21.

**Appendix of Study Protocol**

**Table 1. Search strategy**

| **#1 Database: EMBASE** | |
| --- | --- |
| **Search #** | **Terms** |
| **Population terms** | |
| 1 | exp infant/ |
| 2 | infant*.mp. |
| 3 | exp newborn/ |
| 4 | neonat*.mp. |
| 5 | newborn*.mp. |
| 6 | (baby or babies).mp. |
| 7 | or/1-6 |
| **Concept terms – Hospital-acquired infections** | |
| 8 | exp epidemic/ or exp disease transmission/ |
| 9 | exp cross infection/ |
| 10 | infection*.mp. |
| 11 | sepsis.mp. |
| 12 | septic*.mp. |
| 13 | bacteri?emia.mp. |
| 14 | fung?emia.mp. |
| 15 | exp ventilator associated pneumonia/ or exp catheter infection/ |
| 16 | (central venous catheter or ((central or catheter or device) adj4 infection*) or clabsi).mp. |
| 17 | (pneumonia* or ventilator-associated or ventilator-acquired or vap).mp. |
| 18 | nosocomial*.mp. |
| 19 | outbreak*.mp. |
| 20 | (hospital* adj3 infection*).mp. |
| 21 | (disease* adj3 control*).mp. |
| 22 | epidemic*.mp. |
| 23 | cluster*.mp. |
| 24 | or/8-23 |
| **Concept terms – Care bundles** | |
| 25 | exp care bundle/ |
| 26 | bundle*.mp. |
| 27 | ((set* or multifaceted* or tailored or complex or care) adj3 intervention*).mp. |
| 28 | package*.mp. |
| 29 | checklist*.mp. |
| 30 | (care adj3 pathway*).mp. |
| 31 | or/25-30 |
| **Context terms – LMIC** | |
| 32-322 | LMIC terms |

| 323 | or/32-322 [ALL LOW AND MIDDLE-INCOME COUNTRIES] |
| --- | --- |
| 324 | and/7,24,31,323 |
| 325 | limit 324 to ((English or Spanish or French) and yr=" 2001 -Current") |

| **#2 Database: Pubmed** | |
| --- | --- |
| **Search #** | **Terms** |
| **Population terms** | |
| 1 | (((((((infant[MeSH Terms]) OR infant, newborn[MeSH Terms]) OR infant*[Text Word]) OR newborn*[Text Word]) OR neonat*[Text Word]) OR baby[Text Word]) OR babies[Text Word])) |
| **Concept terms – Hospital-acquired infections** | |
| 2 | ((((((((((((((((((((((((cross infection[MeSH Terms]) OR disease outbreaks[MeSH Terms]) OR disease transmission, infectious[MeSH Terms]) OR infection*[Text Word]) OR sepsis[Text Word]) OR septic*[Text Word]) OR septic shock[Text Word]) OR (bacteremia[Text Word] OR bacteraemia[Text Word])) OR fungemia[Text Word]) OR fungaemia[Text Word]) OR nosocomial*[Text Word]) OR cluster*[Text Word]) OR outbreak*[Text Word]) OR epidemic*[Text Word]) OR "disease control"[Text Word]) OR catheter related infection[MeSH Terms]) OR pneumonia, ventilator associated[MeSH Terms]) OR "central venous catheter"[Text Word]) OR "catheter associated"[Text Word]) OR clabsi[Text Word]) OR pneumonia*[Text  Word]) OR "ventilator associated"[Text Word]) OR "ventilator acquired"[Text Word]) OR vap)) |
| **Concept terms – Care bundles** | |
| 3 | (((((((((patient care bundles[MeSH Terms]) OR bundle*[Text Word]) OR "complex intervention*"[Text Word]) OR "set* of intervention*"[Text Word]) OR "multifaceted intervention*"[Text Word]) OR "care intervention*"[Text Word]) OR package*[Text Word]) OR checklist*[Text Word]) OR "care pathway*"[Text  Word])) |
| **Context terms – LMIC** | |
| 4 | LMIC filter |
| 5 | ((((((((((infant[MeSH Terms]) OR infant, newborn[MeSH Terms]) OR infant*[Text Word]) OR newborn*[Text Word]) OR neonat*[Text Word]) OR baby[Text Word]) OR babies[Text Word])) AND ((((((((((((((((((((((((cross infection[MeSH Terms]) OR disease outbreaks[MeSH Terms]) OR disease transmission, infectious[MeSH Terms]) OR infection*[Text Word]) OR sepsis[Text Word]) OR septic*[Text Word]) OR septic shock[Text  Word]) OR (bacteremia[Text Word] OR bacteraemia[Text Word])) OR fungemia[Text Word]) OR fungaemia[Text Word]) OR nosocomial*[Text Word]) OR cluster*[Text Word]) OR outbreak*[Text Word]) OR epidemic*[Text Word]) OR "disease control"[Text Word]) OR catheter related infection[MeSH Terms]) OR pneumonia, ventilator associated[MeSH Terms]) OR "central venous catheter"[Text Word]) OR "catheter associated"[Text Word]) OR clabsi[Text Word]) OR pneumonia*[Text Word]) OR "ventilator associated"[Text Word]) OR "ventilator acquired"[Text Word]) OR vap)) AND (((((((((patient care bundles[MeSH Terms]) OR bundle*[Text Word]) OR "complex intervention*"[Text Word]) OR "set* of intervention*"[Text Word]) OR "multifaceted intervention*"[Text Word]) OR "care intervention*"[Text Word]) OR package*[Text Word]) OR checklist*[Text Word]) OR "care pathway*"[Text Word])) AND LMIC filter AND 2001/01/01:2020/12/31[Date - Publication]) AND (("English"[Language] OR "French"[Language]) OR "Spanish"[Language]) |

| **#3 Database: CINAHL** | |
| --- | --- |
| **Search #** | **Terms** |
| **Population terms** | |
| S1 | (MH "Infant, Newborn, Diseases+") OR (MH "Infant, Newborn+") |
| S2 | infant* or neonat* or newborn* or baby or babies |
| S3 | S1 OR S2 |
| **Concept terms – Hospital-acquired infections** | |
| S4 | (MH "Cross Infection+") |
| S5 | (MH "Disease Transmission+") OR (MM "Disease Transmission, Professional-to-Patient") OR (MH "Disease Transmission, Horizontal+") |
| S6 | (MM "Disease Outbreaks") |

| S7 | (MH "Catheter-Related Infections+") OR (MM "Catheter-Related Bloodstream Infections") OR (MM "Urinary Tract Infections, Catheter-Related") |
| --- | --- |
| S8 | (MM "Pneumonia, Ventilator-Associated") |
| S9 | infection* or sepsis or septic* bacteri?emia or fung?emia or nosocomial* or outbreak* or epidemic* or cluster* or "central venous catheter" or "central associated" or "device-associated" or clabsi or pneumonia* or "ventilator-associated" or "ventilator-acquired" or vap or (hospital* N3 infection*) or (disease* N3 control*) |
| S10 | S4 OR S5 OR S6 OR S7 OR S8 OR S9 |
| **Concept terms – Care bundles** | |
| S11 | (MH "Continuity of Patient Care+") OR (MH "Patient Care+") |
| S12 | bundle* or package* or checklist* or (care W3 pathway*) or ((set* or multifaceted* or tailored or complex or care) W3 intervention*) |
| S13 | S11 OR S12 |
| **Context terms – LMIC** | |
| S14-S31 | LMIC terms |
| S32 | LMIC terms combined with OR |
| S33 | S3 AND S10 ANDS13 AND S32 |

| **#4 Database: Global Health** | |
| --- | --- |
| **Search #** | **Terms** |
| **Population terms** | |
| 1 | exp infants/ |
| 2 | infant*.mp. |
| 3 | exp neonates/ |
| 4 | neonat*.mp. |
| 5 | newborn*.mp. |
| 6 | (baby or babies).mp. |
| 7 | or/1-6 |
| **Concept terms – Hospital-acquired infections** | |
| 8 | exp disease transmission/ or outbreaks.sh. |
| 9 | Cross infection/ |
| 10 | infection*.mp. |
| 11 | sepsis.mp. |
| 12 | septic*.mp. |
| 13 | bacteri?emia.mp. |
| 14 | fung?emia.mp. |
| 15 | exp ventilator associated pneumonia/ or bloodstream infections/ |
| 16 | (central venous catheter or ((central or catheter or device) adj4 infection*) or clabsi).mp. |
| 17 | (pneumonia* or ventilator-associated or ventilator-acquired or vap).mp. |
| 18 | nosocomial*.mp. |
| 19 | outbreak*.mp. |

| 20 | (hospital* adj3 infection*).mp. |
| --- | --- |
| 21 | (disease* adj3 control*).mp. |
| 22 | epidemic*.mp. |
| 23 | cluster*.mp. |
| 24 | or/8-23 |
| **Concept terms – Care bundles** | |
| 25 | exp disease control/ |
| 26 | bundle*.mp. |
| 27 | ((set* or multifaceted* or tailored or complex or care) adj3 intervention*).mp. |
| 28 | package*.mp. |
| 29 | checklist*.mp. |
| 30 | (care adj3 pathway*).mp. |
| 31 | or/25-30 |
| **Context terms – LMIC** | |
| 32-322 | LMIC terms |
| 323 | or/32-322 [ALL LOW AND MIDDLE-INCOME COUNTRIES] |
| 324 | and/7,24,31,323 |
| 325 | limit 324 to ((english or spanish or french) and yr=" 2001 -Current") |

**#5 Database: Web of Science**

**Search # Population terms** #1

**Terms**

TS=(infant*) OR TS=(neonat*) OR TS=(newborn*) OR TS=(baby) OR TS=( babies)

**Concept terms – Hospital-acquired infections**

#2 TS=(infection*) OR TS=(sepsis) OR TS=(septic*) OR TS=(bacteri$emia) OR TS=(fung$emia) OR TS=(nosocomial*) OR TS=(outbreak*) OR TS=(epidemic*) OR TS=(cluster*) OR TS=(clabsi) OR TS=("cross-infection")

#3 **TOPIC:** ("central venous catheter") *OR* **TOPIC:** ((central or catheter device) NEAR/4 infection*) *OR* **TOPIC:** (clabsi) *OR* **TOPIC:** (pneumonia*) OR **TOPIC:** ("ventilator-associated") *OR* **TOPIC:** ("ventilator-associated pneumonia") *OR* **TOPIC:** ("ventilator associated pneumonia") *OR*

TOPIC: ("ventilator-acquired") *OR* **TOPIC:** (vap) *OR* **TOPIC:** (hospital* NEAR/3 infection*) *OR* **TOPIC:** (disease* NEAR/3 control*)

#4 #3 OR #4

**Concept terms – Care bundles**

#5 **TOPIC:** (bundle*) *OR* **TOPIC:** (package*) *OR* **TOPIC:** (checklist*)

*OR* **TOPIC:** (care NEAR/3 pathway*) *OR* **TOPIC:** ((set* or multifaceted or tailored or complex or care) NEAR/3 intervention*) *OR* **TOPIC:** (patient NEAR/3 bundle*)

**Context terms – LMIC**

#6 LMIC terms

#1 AND #4 AND #5 AND #6

**Refined by: PUBLICATION YEARS:** (2020 OR 2016 OR 2013 OR 2006 OR 2019 OR 2015 OR 2012 OR 2017 OR 2014 OR 2010 ) AND **LANGUAGES:** ( ENGLISH )

#7

**Table 2. Definitions of implementation strategies used, proposed by the ERIC.**

| **Strategy** | **Definitions** |
| --- | --- |
| **Audit and provide feedback** | Collect and summarise clinical performance data over a specified time period and give it to clinicians and administrators to monitor, evaluate, and modify provider behaviour |
| **Change physical structure and equipment** | Evaluate current configurations and adapt, as needed, the physical structure and/or equipment (e.g., changing the layout of a room, adding equipment) to best accommodate the targeted innovation |
| **Conduct educational meetings** | Hold meetings targeted toward different stakeholder groups (e.g., providers, administrators, other organisational stakeholders, and community, patient/consumer, and family stakeholders) to teach them about the clinical innovation |
| **Create new clinical teams** | Change who serves on the clinical team, adding different disciplines and different skills to make it more likely that the clinical innovation is delivered (or is more successfully delivered) |
| **Create or change credentialing and/or licensure standards** | Create an organisation that certifies clinicians in the innovation or encourage an existing organisation to do so. Change governmental, professional certification or licensure requirements to include delivering the innovation. Work to alter continuing education requirements to shape professional practice toward the innovation |
| **Develop educational materials** | Develop and format manuals, toolkits, and other supporting materials in ways that make it easier for stakeholders to learn about the innovation and for clinicians to learn how to deliver the clinical innovation |
| **Organise clinician implementation team meetings** | Develop and support teams of clinicians who are implementing the innovation and give them protected time to reflect on the implementation effort, share lessons learned, and support one another's learning |
| **Recruit, designate, and train for leadership** | Recruit, designate, and train leaders for the change effort |
| **Remind clinicians** | Develop reminder systems designed to help clinicians to recall information and/or prompt them to use the clinical innovation |
| **Revise professional roles** | Shift and revise roles among professionals who provide care and redesign job characteristics |

**Figure 1. Algorithm for the classification of quantitative study designs (NICE).**


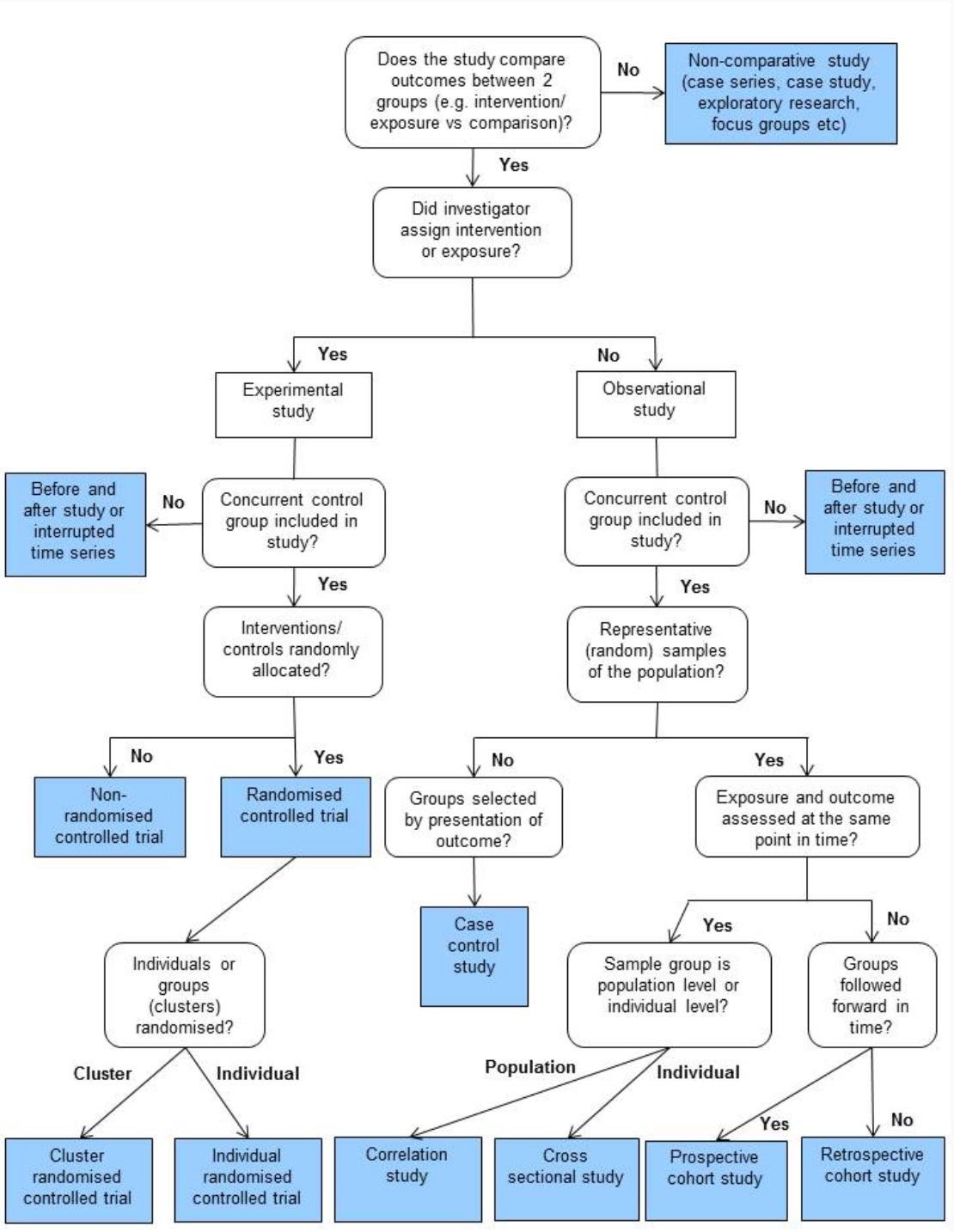


**Figure 2. Cochrane EPOC definitions of study designs.**


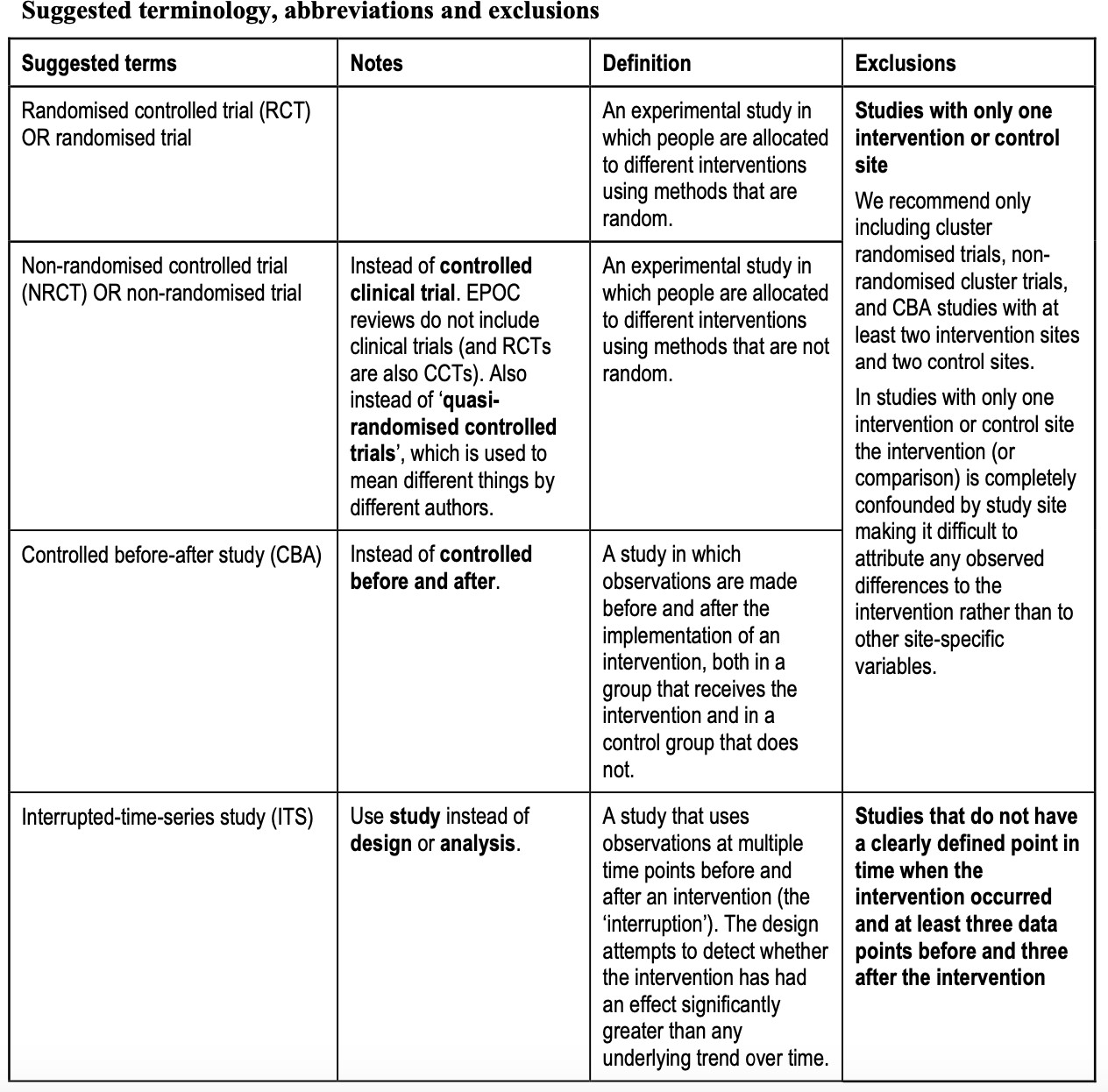


# Figure 2. Qualitative inductive content analysis methodology for the construction of the Classification Framework for the care bundles elements

**1^st^ step: Preparation**

**2^nd^ step: Organisation**

**3^rd^ step: Reporting**

- Labelling of each care bundle element with coding headings to summarise their meaning
- Collating coding headings with similar meaning under higher order headings to develop groups and subgroups through their repeated examination and comparison*
- Naming and providing a definition for groups and subgroups based on the information they contained
- Categorising the infection prevention and care bundles into groups
- Identification and extraction of infection prevention and care bundles in the published literature
- Bundle element extraction (i.e. extraction of the single interventions that make up each of the care bundles identified).
- Familiarisation with bundle element data.

* If the headings of the bundle elements were related to implementation strategies, these were grouped after the categories created by Powell and colleagues in the Expert Recommendations for Implementing Change (ERIC) study.

- Quantitative analysis (frequencies and percentages) performed on the content of the groups and subgroups categories of the Classification Framework

30
